# Fractionated vs single-dose gemtuzumab ozogamicin with determinants of benefit in older patients with AML: the UK NCRI AML18 trial

**DOI:** 10.1182/blood.2023020630

**Published:** 2023-08-22

**Authors:** Sylvie D. Freeman, Abin Thomas, Ian Thomas, Robert K. Hills, Paresh Vyas, Amanda Gilkes, Marlen Metzner, Niels Asger Jakobsen, Alison Kennedy, Rachel Moore, Nuria Marquez Almuina, Sarah Burns, Sophie King, Georgia Andrew, Kathleen M. E. Gallagher, Rob S. Sellar, Paul Cahalin, Duruta Weber, Mike Dennis, Priyanka Mehta, Steven Knapper, Nigel H. Russell

**Affiliations:** 1 Institute of Immunology and Immunotherapy, University of Birmingham, Birmingham, United Kingdom; 2 Centre for Trials Research, Cardiff University, Cardiff, United Kingdom; 3 Nuffield Department of Population Health, University of Oxford, Oxford, United Kingdom; 4 Weatherall Institute of Molecular Medicine, University of Oxford, Oxford, United Kingdom; 5 Cardiff University School of Medicine, Cardiff, United Kingdom; 6 Wellcome, Medical Research Council Cambridge Stem Cell Institute, University of Cambridge, Cambridge, United Kingdom; 7 Laboratory of Myeloid Malignancies, National Heart, Lung, and Blood Institute, Bethesda, MD; 8 Cellular Immunotherapy Program, Massachusetts General Hospital Cancer Center, Harvard Medical School, Boston, MA; 9 UCL Cancer Institute and University College London Hospital, London, United Kingdom; 10 Blackpool Teaching Hospitals National Health Service Foundation Trust, Blackpool, United Kingdom; 11 Odense University Hospital, Odense, Denmark; 12 The Christie National Health Service Foundation Trust, Manchester, United Kingdom; 13 The University of Bristol and Weston National Health Service Trust, Bristol, United Kingdom; 14 Guy's and St Thomas' National Health Service Foundation Trust, London, United Kingdom

## Abstract

Addition of gemtuzumab ozogamicin (GO) to induction chemotherapy improves outcomes in older patients with acute myeloid leukemia (AML), but it is uncertain whether a fractionated schedule provides additional benefit to a single dose. We randomized 852 older adults (median age, 68-years) with AML/high-risk myelodysplasia to GO on day 1 (GO1) or on days 1 and 4 (GO2) of course 1 induction. The median follow-up period was 50.2 months. Although complete remission (CR) rates after course 1 did not significantly differ between arms (GO2, 63%; GO1, 57%; odds ratio [OR], 0.78; *P* = .08), there were significantly more patients who achieved CR with a measurable residual disease (MRD)<0.1% (50% vs 41%; OR, 0.72; *P* = .027). This differential MRD reduction with GO2 varied across molecular subtypes, being greatest for *IDH* mutations. The 5-year overall survival (OS) was 29% for patients in the GO2 arm and 24% for those in the GO1 arm (hazard ratio [HR], 0.89; *P* = .14). In a sensitivity analysis excluding patients found to have adverse cytogenetics or *TP53* mutations, the 5-year OS was 33% for GO2 and 26% for GO1 (HR, 0.83; *P* = .045). In total, 228 (27%) patients received an allogeneic transplantation in first remission. Posttransplant OS was superior in the GO2 arm (HR, 0.67; *P* = .033); furthermore, the survival advantage from GO2 in the sensitivity analysis was lost when data of patients were censored at transplantation. In conclusion, GO2 was associated with a greater reduction in MRD and improved survival in older adults with nonadverse risk genetics. This benefit from GO2 was dependent on allogeneic transplantation to translate the better leukemia clearance into improved survival. This trial was registered at www.isrctn.com as #ISRCTN 31682779.

## Key Points

• Fractionated compared with single-dose gemtuzumab increased response depth across most AML molecular groups without increasing toxicity.
• Older patients with AML receiving fractionated gemtuzumab had improved survival when induction was consolidated by allograft.

## Visual Abstract

**Figure:**
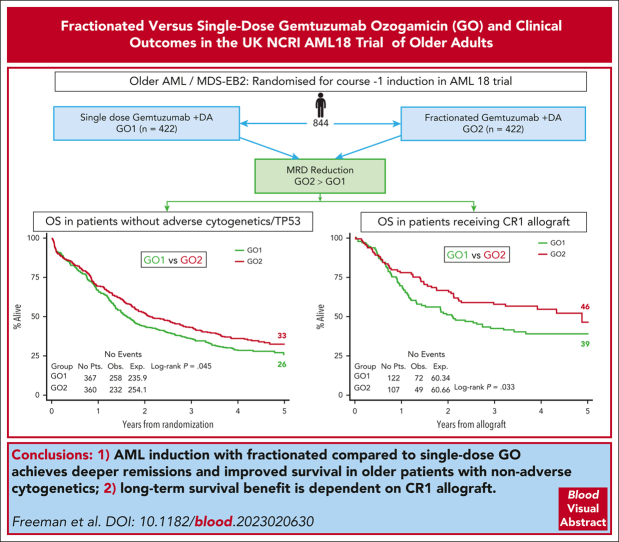

## Teaser

The conjugated anti-CD33 monoclonal antibody, gemtuzumab ozogamicin (GO), is highly active against acute myeloid leukemia (AML), but dosing and scheduling issues have bedeviled its stable integration into routine clinical care regimens. In this month’s CME article, Freeman and colleagues present the mature results of a large trial showing for the first time in a randomized comparison that the addition of 2 fractionated doses of GO to intensive induction chemotherapy is better than 1 in patients with intermediate- and good-risk AML, including in those patients undergoing allogeneic transplantation.

**Medscape Continuing Medical Education online**

In support of improving patient care, this activity has been planned and implemented by Medscape, LLC and the American Society of Hematology. Medscape, LLC is jointly accredited with commendation by the Accreditation Council for Continuing Medical Education (ACCME), the Accreditation Council for Pharmacy Education (ACPE), and the American Nurses Credentialing Center (ANCC), to provide continuing education for the healthcare team.

Medscape, LLC designates this Journal-based CME activity for a maximum of 1.0 *AMA PRA Category 1 Credit(s)*™. Physicians should claim only the credit commensurate with the extent of their participation in the activity.

Successful completion of this CME activity, which includes participation in the evaluation component, enables the participant to earn up to 1.0 MOC points in the American Board of Internal Medicine's (ABIM) Maintenance of Certification (MOC) program. Participants will earn MOC points equivalent to the amount of CME credits claimed for the activity. It is the CME activity provider's responsibility to submit participant completion information to ACCME for the purpose of granting ABIM MOC credit.

All other clinicians completing this activity will be issued a certificate of participation. To participate in this journal CME activity: (1) review the learning objectives; (2) study the education content; (3) take the post-test with a 75% minimum passing score and complete the evaluation at https://www.medscape.org/journal/blood; and (4) view/print certificate. For CME questions, see page 1759.

**Disclosures**

CME questions author Laurie Barclay, freelance writer and reviewer, Medscape, LLC, declares no competing financial interests.

**Learning Objectives**

Upon completion of this activity, participants will:

1. Describe survival, response, and toxicity outcomes of single vs fractionated (2 doses given on days 1 and 4) gemtuzumab ozogamicin (GO) dosing in the first induction course, based on the randomized NCRI AML18 trial of older adults with acute myeloid leukemia (AML)
2. Describe differential efficacy between the GO schedules across molecular subgroups, based on molecular profiling together with flow cytometric measurable residual disease testing, and other factors affecting outcomes among older adults with AML enrolled in the randomized NCRI AML18 trial
3. Identify clinical implications of outcomes of single vs fractionated GO dosing and of factors affecting outcomes, based on the randomized NCRI AML18 trial of older adults with AML

Release date: November 16, 2023; Expiration date: November 16, 2024

## Introduction

Currently, available treatment with a combination of daunorubicin and cytosine arabinoside (Ara-C) has achieved a remission rate of >60% in patients aged >60 years with acute myeloid leukemia (AML) considered fit for intensive treatment. However, approximately three-quarters of these patients relapse within 3 years. In the National Cancer Research Institute (NCRI) AML16 trial for older adults (median age, 67 years), we previously reported that the addition of a single dose of gemtuzumab ozogamicin (GO) to 2 different induction therapies, daunorubicin/Ara-C (DA) and daunorubicin/clofarabine, was found to improve overall survival (OS; 20% vs 15% at 4 years; hazard ratio [HR], 0.82 [0.72-1.0]; *P* = .05) because of a reduction in relapse risk.^1^ This was not associated with any increased hematologic or nonhematologic toxicity. In a second study, ALFA 0701, the ALFA (Acute French Leukemia Association) group reported that the addition of a fractionated schedule of 3 doses of GO to DA induction chemotherapy for patients aged from 50 to 70 years also significantly improved the event-free survival, leading to regulatory approval; they observed no increase in induction deaths, but hematologic toxicity was augmented, particularly with regard to platelet recovery.^2^^,^^3^ GO was also given at consolidation in this ALFA trial. These 2 studies demonstrate that the addition of GO to standard chemotherapy improves outcomes in older patients with AML. Although direct comparison of the 2 trials is difficult because of different age ranges, there is some suggestion that the fractionated GO schedule used in the ALFA 0701 trial gave a greater survival benefit than the single dose used in AML16, albeit with increased toxicity.^4^ In the NCRI AML18 trial, we randomized a single vs a fractionated schedule of GO in the first induction course to address the question of whether fractionated dosing provides a survival advantage in older adults. The fractionated schedule used 2 doses of GO administered on days 1 and 4 rather than the 3-dose schedule used in the ALFA 0701 study because of concerns over toxicity, particularly delayed platelet count recovery. Owing to the previously observed lack of benefit of GO in the adverse cytogenetic risk group,^4^ patients were excluded from the randomization if they were known to have adverse cytogenetics before trial entry, but awaiting cytogenetic results was not mandated for trial entry. Here, we report the 5-year outcomes from this trial. In addition to cytogenetics, specific gene mutations have been reported to predict the benefit from GO through a post hoc analysis of the ALFA 0701 study.^5^ Molecular profiling together with flow cytometric measurable residual disease (MRD) testing were performed in AML18 to evaluate for any differential efficacy between the GO schedules across molecular subgroups.

## Methods

### Patients and trial treatments

The AML18 protocol (ISRCTN-31682779; EudraCT-2013-002730-21) was designed for older patients aged ≥60 years who were fit for intensive chemotherapy and did not have blast transformation of chronic myeloid leukemia or acute promyelocytic leukemia. Patients with high-risk myelodysplastic syndrome, which was defined as >10% marrow blasts at diagnosis, were eligible. The protocol permitted patients (n = 23) aged <60 years, who were not considered suitable for the concurrent NCRI AML19 trial for younger patients (which included high-dose Ara-C) to enter after discussion with a trial coordinator. Clinical secondary AML was defined as resulting from either antecedent hematologic disorder or prior chemotherapy for a nonhematologic malignancy. Patients were randomly assigned to receive either 1 (GO1: 3 mg/m^2^ on day 1, dose not capped) or 2 doses of GO (GO2: given on days 1 and 4; 3 mg/m^2^, with a maximum of 5 mg per dose), with induction chemotherapy comprising daunorubicin (60 mg/m^2^ on days 1, 3, and 5) and Ara-C (100 mg/m^2^ IV twice daily on days 1-10). To be eligible for randomization, patients were required to have serum alanine aminotransferase, aspartate aminotransferase ≤2.5 × upper limit of normal and bilirubin ≤2.0 × upper limit of normal. Patients with known adverse cytogenetics were ineligible; however, cytogenetic results were not a requirement for trial entry. Further courses of chemotherapy did not include GO. After the first course, the second course assignment was dependent on response status that included measurement of MRD status by bone marrow assessment. Patients in complete remission (CR) or CR with incomplete hematologic recovery (CRi) and categorized as having an MRD-negative status received a second course of DA (daunorubicin 50 mg/m^2^ × 3 and Ara-C 100 mg/m^2^ twice daily × 8 days), followed by a course of intermediate-dose cytarabine (1 mg/m^2^ × 5 days). Patients who after the first course did not attain a CR or CRi or were in remission but categorized as having MRD-positive status or unassessable were eligible for randomization between DA or intensification with either FLAG-Ida (fludarabine, Ara-C, G-CSF with idarubicin) adjusted for age or DA plus cladribine. Independent of response status after the first or second course, all patients were eligible for a nonintensive (reduced intensity conditioning) allogeneic stem transplant if a suitable HLA-matched donor was available.

Patients were enrolled from 81 centers in the United Kingdom and 6 in Denmark. The study was approved by the ethics committees (Wales MultiCentre Research Ethics Committee together with national and regional ethics bodies in Denmark for sites in Denmark) and conducted in accordance with Good Clinical Practice guidelines and the Declaration of Helsinki. All patients provided written informed consent.

### Laboratory studies

Cytogenetic analyses, performed locally, were reviewed and coded centrally according to the Grimwade 2010 criteria.^6^ Mutation analysis of *FLT3* and *NPM1* was performed in a single reference laboratory. Banked diagnostic DNA was analyzed for variants in 95 recurrently mutated myeloid genes (supplemental Methods, available on the *Blood* website). AML with secondary-type mutations (myelodysplasia-related mutations) was defined by presence of ≥1 mutations in *ASXL1*, *BCOR*, *EZH2*, *RUNX1*, *SF3B1*, *SRSF2*, *STAG2*, *U2AF1*, or *ZRSR2.*^7^, ^8^, ^9^ For the ALFA 1200 risk score,^10^ patients were classified as “no-go” (poor) if they had poor risk cytogenetics (per European LeukemiaNet [ELN] 2017 guidelines^11^) and either a mutation in *KRAS* or *TP53*; “go-go” (favorable) if they had non–poor risk cytogenetics and either wild-type *NPM1*, *FLT3-*ITD, *DNMT3A*, *ASXL1*, and *NRAS* or mutated *NPM1* and ≤1 mutation in *FLT3-ITD* (only low AR), *DNMT3A*, *ASXL1*, and *NRAS;* and “slow-go” (intermediate) consisted of all other patients.

MRD was assessed by flow cytometry at a single reference laboratory as previously described.^12^, ^13^, ^14^ Details of sample logistics, processing, and analysis strategy are provided in supplemental Methods. Results were entered into the trial database within 24 to 48 hours of sample receipt, blinded to the investigator-reported remission status. Patients were categorized as being “MRD unassessable” if no adequate bone marrow sample was received before course 2 assignment. Flow cytometric MRD testing combined the detection of diagnostic leukemic aberrant immunophenotypes (LAIPs) and different-from-normal aberrant immunophenotypes, as per consensus recommendations,^15^ with any measurable level of MRD considered positive (above a sensitivity threshold of 0.02%-0.05%). An MRD-negative result required negativity in an adequate bone marrow by both different-from-normal and LAIP analysis (prerequisite of LAIP target[s] identified at baseline).

### Statistical considerations and end points

The primary end point of the GO randomization was OS. In the ALFA 0701 study,^2^ 3-year survival on fractioned GO was ∼50% compared with only >30% in the control arm. This was a younger population than those in AML16,^1^ in which 3-year survival was 25% for single-dose GO and 20% for chemotherapy alone. Allowing for 10% of patients who would not fulfill the hepatic entry criteria for GO, it was anticipated that the GO comparison would require no more than 800 patients entered into AML18 to detect, with 80% power at *P* < .05, a 10% difference in survival from 25% to 35%, equivalent to an HR of 0.76, with a critical number of 412 deaths.

The analyses are based on intention to treat, unless otherwise stated. End points were defined according to the revised International Working Group criteria.^16^ Responses were based on investigator assessment of bone marrows. CR and CRi (up to 50 days for course 1 response and up to 100 days for induction response) have been combined for outcome analyses. All outcomes were summarized at 5 years of follow-up. Toxicity (hematologic recovery times and nonhematological toxicity) was scored using the National Cancer Institute Common Toxicity Criteria, version 3, and resource use data (blood product support, days on antibiotics, and hospitalization) were collected.

Characteristics of the patients are summarized across the group using frequency and percentage for categorical data, and median and quartile range for quantitative data. Comparisons of patient characteristics were made using χ^2^ tests, Mantel-Haenszel tests for trend, or Wilcoxon rank-sum tests, as appropriate. Time-to-event outcomes were compared using log-rank tests and Cox regression. Outcomes are reported as effect sizes with 95% confidence intervals (CIs); significance was set at *P* < .05. For the exploratory analyses of key subgroups with forest plots, HRs were calculated using Cox proportional hazards models, with a test for trend of heterogeneity across the subgroups, wherever applicable. For the comparison of transplant vs no transplant, to counteract the immortal time bias introduced by patients needing to have survived long enough to receive a transplant, Mantel-Byar methodology was used.

## Results

### Patient characteristics

**Figure 1.**
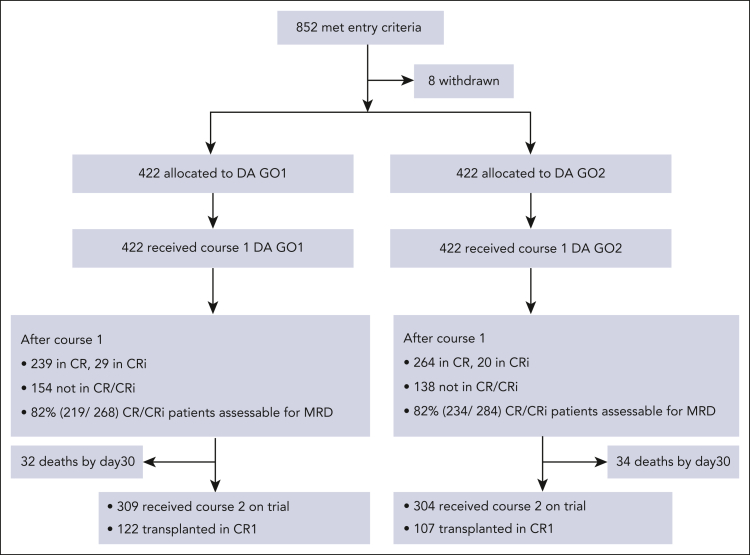
**Consolidated Standards of Reporting Trials diagram.**

Between 4 November 2014 and 12 March 2019, 852 patients with AML or high-risk myelodysplastic syndrome (EB2) were randomly assigned to GO1 or GO2 of gemtuzumab combined with course 1 induction chemotherapy (“Methods”; Figure 1). The median follow-up time was 50.2 months (quartiles, 44-60). Data were unavailable for 8 patients who withdrew trial consent. The median age of patients was 68 years, with 33.5% aged between 70 and 81 years. Because many patients in this study were randomized before their cytogenetic results, the cohort eventually included 117 (14%) with adverse cytogenetics or TP53-mutated AML. Clinical and genetic baseline characteristics of the patients are shown in Table 1 and were balanced between the treatment arms. In total, 747 patients had mutation panel data and could therefore also be categorized based on the ALFA 1200 genomic score for older adults. In the AML18 cohort, distribution between the ALFA 1200 genetic subgroups (validated as a prognostic score for older patients receiving intensive chemotherapy)^10^ was comparable with that of the reported ALFA cohort, with 52% categorized as slow-go. Moreover, 48% of the patients had AML with secondary-type mutations (myelodysplasia-related mutations).^7^, ^8^, ^9^

**Table 1.** **Patient demographics and clinical characteristics**

	Overall	GO1	GO2
	N = 844	n = 422	n = 422
**Age, y, median (range)**	68 (50-81)	67.5 (51-79)	68 (50-81)
Age ≥65 y	606 (72%)	301 (71%)	305 (72%)
Age ≥70 y	283 (34%)	141 (33%)	142 (34%)
Male	511 (61%)	256 (61%)	255 (60%)
**WBC ×10**^**9**^**/L median (range)**	5.8 (0.3-416.6)	6.25 (0.3-416.6)	5.65 (0.4-365)
<10	493 (58%)	247 (59%)	246 (58%)
≥50	101 (12%)	50 (12%)	51 (12%)
**Diagnosis**			
Clinical de novo AML	673 (80%)	338 (80%)	335 (79%)
Clinical secondary AML	89 (11%)	44 (10%)	45 (11%)
High-risk MDS	82 (10%)	40 (10%)	42 (10%)
**Performance ID (ECOG**)			
0	404 (48%)	202 (48%)	202 (48%)
1	386 (46%)	193 (46%)	193 (46%)
2	54 (6%)	27 (6%)	27 (6%)
**Genetic risk**			
Cytogenetic (Grimwade 2010)			
Favorable	30 (4%)	22 (6%)	8 (2%)
Intermediate	558 (81%)	277 (79%)	281 (82%)
Adverse	103 (15%)	50 (14%)	53 (16%)
Failed	56	25	31
Not reported	97	48	49
*TP53*^+^	69 (9%)	32 (9%)	37 (10%)
**ELN 2017**			
Favorable	236 (33%)	121 (33%)	115 (32%)
Intermediate	166 (23%)	85 (23%)	81 (23%)
Adverse	323 (45%)	160 (44%)	163 (45%)
Unknown	119	56	63
**ALFA 1200**∗			
Go-go (favorable)	274 (40%)	140 (40%)	134 (39%)
Slow-go (intermediate)	360 (52%)	180 (52%)	180 (53%)
No-go (unfavorable)	57 (8%)	29 (8%)	28 (8%)
Unknown	153	73	80

ECOG, Eastern Cooperative Oncology Group; ALFA, Acute Leukemia French Association; MDS, myelodysplastic syndrome; WBC, white blood cell count.

∗ Details of ALFA 1200 genetic score classification^10^ are provided in “Methods.”

### Response and outcome

**Figure 2.**
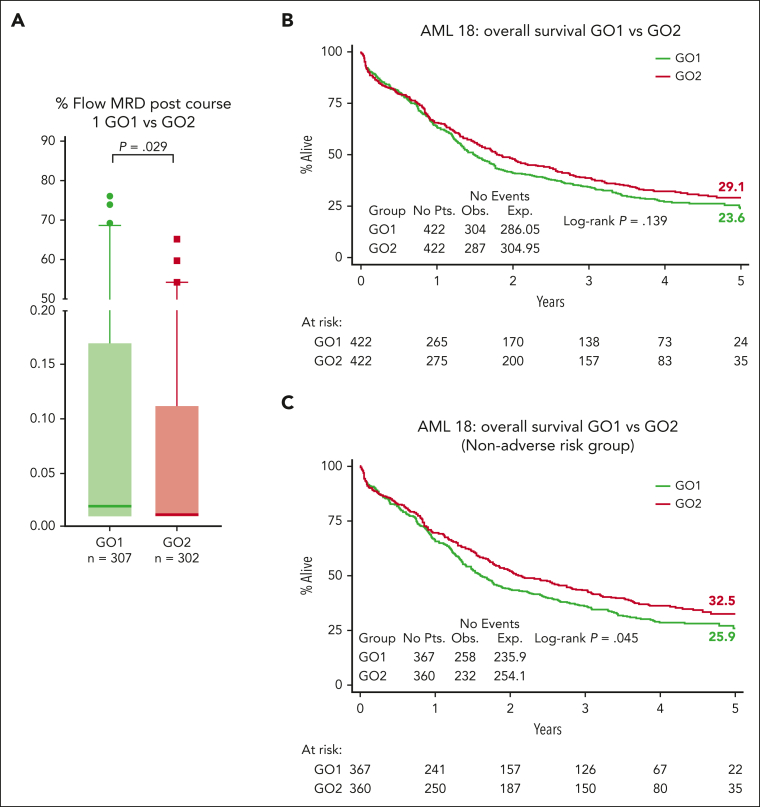
**MRD response and survival outcomes.** (A) Flow cytometric MRD levels in bone marrow after course 1 according to treatment arm. Median levels, presented as a percentage, were significantly lower in the GO2 arm (median, 25%-75% quartiles with 1-99 percentiles are shown). Comparisons were performed using the Mann-Whitney *U* test for continuous variables. Patients with MRD <0.1% were 72% (218 of 302) in the GO2 arm and 62% (191 of 307) in the GO1 arm. Patients with undetectable MRD were 57% (173 of 302) in the GO2 arm and 49.5% (152 of 307) in the GO1 arm. Results represent all patients with MRD data (including those not in CR/CRi after course 1). (B) OS. (C) OS of patients without adverse cytogenetics or mutated *TP53.*

There was no significant difference between GO1 and GO2 for overall response rates (CR and CRi) and CR rates either after the first induction course (64% vs 67% for CR/CRi [*P* = .247]; 57% vs 63% for CR [*P* = .079]) or after 100 days from randomization (82% vs 81% for CR/CRi [*P* = .723]; 73% vs 72% for CR [*P* = .939]; Table 2). Next, we assessed the impact of GO2 vs GO1 on MRD reduction measured by flow cytometry. MRD response data were available for 609 patients after course 1 (GO1, 307; GO2, 302), including 453 patients (74%) who were in CR/CRi (Figure 1; supplemental Figure 1A). Absence of MRD data was associated with worse performance status and older age (supplemental Table 1). Patients with missing MRD results (GO1, 115; GO2, 120) had a low post–course 1 overall response rate (42%; 99 of 235), but between treatment arms, clinical response profiles were equivalent (supplemental Figure 1). Among the patients who were evaluable for response by flow cytometric MRD, lower MRD levels were observed in patients assigned to GO2 than in those assigned to GO1 (*P* = .029; Figure 2A). Of the randomized patients, 745 were assessable for composite response that included MRD for those in CR and CRi after first induction (Table 2). Among these 745 patients, CR without MRD based on ELN criteria (MRD <0.1%) was achieved by 50% and 41% of patients assigned to GO2 and GO1, respectively (odds ratio [OR], 1.39 [95% CI, 1.04-1.86]; *P* = .025; Table 2).

**Table 2.** **Response, early deaths, and rates of ASCT**

	GO1	GO2	*P* value	OR (95% CI)
	n = 422 (%)	n = 422 (%)		
**Response**				
CR + CRi	346 (82)	342 (81)	.723	1.06 (0.75-1.52)
CR	306 (73)	305 (72)	.939	1.01 (0.75-1.37)
CRi	40 (10)	37 (9)	.720	1.1 (0.68-1.75)
CR after course 1	239 (57)	264 (63)	.079	0.78 (0.59-1.03)
CR + CRi after course 1	268 (64)	284 (67)	.247	0.85 (0.64-1.12)
**Response including MRD status after course 1**	N = 373 (%)∗	N = 372 (%)∗		
CR + CRi	219 (59)	234 (63)	.241	0.84 (0.63-1.12)
CR + CRi MRD <0.1%†	171 (46)	194 (52)	.085	0.78 (0.58-1.03)
CR MRD <0.1%†	154 (41)	184 (50)	.025	0.72 (0.54-0.96)
CR + CRi MRD negative	144 (39)	158 (43)	.282	0.85 (0.64-1.14)
CR MRD negative	130 (35)	152 (41)	.091	0.78 (0.57-1.04)
**Early death**				
Day 30	32 (8)	34 (8)	.797	0.94 (0.56-1.54)
Day 60	44 (10)	52 (12)	.386	0.83 (0.54-1.27)
**ASCT**	144 (34)	128 (30)	.239	1.19 (0.89-1.59)
Allograft in CR1	122 (29)	107 (25)	.215	1.22 (0.89-1.64)
Time to allograft in CR1. median (range) days‡	108 (0-462)	111 (16-342)	.349	—

χ^2^ or exact test used to generate the *P* values. MRD measured by flow cytometry.

OR, odds ratio.

∗ All patients not attaining CR/CRi (including day 30 deaths) + patients in CR/CRi with MRD data.

† MRD <0.1%, MRD negative or detectable but <0.1%.

‡ Wilcoxon rank-sum test is used to generate the *P* value.

The 5-year OS was 24% in patients assigned to GO1 and 29% in patients assigned to GO2 (HR, 0.89 [95% CI, 0.75-1.04]; *P* = .14; Figure 2A). The 5-year event-free survival was 19% for the GO1 treatment group vs 21% for GO2 (HR, 0.94 [95% CI, 0.81-1.09]; *P* = .422).

In a sensitivity analysis that excluded patients with adverse cytogenetics/*TP53*-mutated AML, there was a significant differential improvement in the OS rate for GO2, with 5-year OS estimates of 33% vs 26% for GO1 (HR, 0.83 [95% CI, 0.70-1.00]; *P* = .045; Figure 2B) but not for event-free survival (24% vs 20% for GO1; HR, 0.88 [95% CI, 0.74-1.04]; *P* = .14). GO2 was also associated with higher relapse free survival (RFS) at 3 years in patients without adverse genetics, although this did not reach significance, RFS at 3 years, 39% vs 32% for GO1; HR, 0.81 [95% CI, 0.65-1.01]; *P* = .062; RFS at 5 years, 29% vs 26% for GO1; HR, 0.84 [95% CI, 0.68-1.05]; *P* = .12).

The results for OS at 5 years from the time of randomization favored GO2 in most subgroups based on clinically defined baseline characteristics (age, white blood cell count, disease type, and cytogenetics). However, there was a significant interaction based on age, indicating a lack of benefit from GO2 compared with a single dose of GO for patients aged >70 years (supplemental Figure 2).

**Figure 3.**
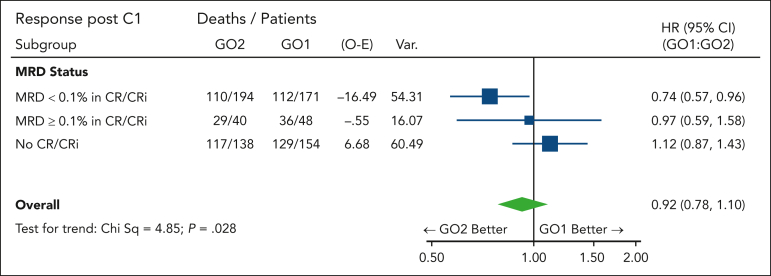
**Forest plot for OS according to the treatment arm stratified based on residual disease response after course 1.** MRD <0.1%, MRD negative or detectable but <0.1%.

MRD-measured response may potentially serve as a surrogate end point because of its association with long-term survival. In addition to the better MRD clearance observed in the GO2 group, the survival benefit from GO2 was most apparent in patients with leukemia reduction to an MRD of <0.1% after course 1 (HR, 0.74 [95% CI, 0.57-0.96]; Figure 3; positive test for trend; *P* = .028). We hypothesized that the differential reduction in leukemia burden could extend below flow cytometric MRD assay detection limits, with a consequent effect on survival in patients categorized as having an MRD-negative status. For these patients, OS at 5 years was 41% in the GO2 arm, as compared with 28% in the GO1 arm (HR, 0.76 [95% CI, 0.572-0.999]; *P* = .050), supportive of a correlation between MRD response and survival.

### Response and outcome based on molecularly defined subtype

**Figure 4.**
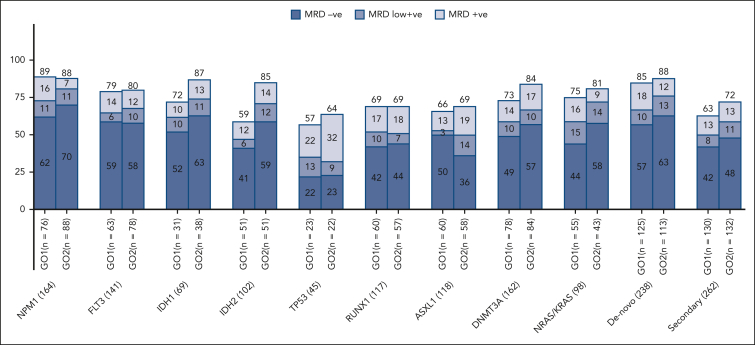
**Residual disease response rates by molecular subtypes per treatment arm.** MRD negative, MRD not detectable; MRD low positive, MRD detected but <0.1%; MRD positive, MRD ≥0.1% (ELN flow cytometry MRD threshold).

Remission rates varied between examined molecular subtypes after the first course of standard chemotherapy combined with GO, but between treatment arms, a differential improvement from that of GO2 was observed in patients with *IDH1* mutations (CR/CRi of 78% vs 66% with GO1) and *IDH2* mutations (CR/CRi of 71% vs 53% with GO1; supplemental Figure 3). When response depth was evaluated by flow cytometric MRD status in patients with post–course 1 results, the frequencies of patients achieving MRD-negative remissions were higher by >10% in the GO2 group compared with that in the GO1 group for the mutational subtypes of *IDH1* and *IDH2* (Figure 4).

We examined for possible differential effects on OS according to the treatment arm in an exploratory analysis of molecularly defined AML subtypes. Survival advantage from GO2 vs GO1 was most apparent for the subgroup of patients with DNA methylation–type mutations (mutated *IDH* 1 or 2 and *DNMT3A*; HR, 0.77 [95% CI, 0.63-0.93]; supplemental Figure 4A). Patients with *IDH1* or *IDH2* mutations at baseline randomized to GO2 had a 5-year OS of 31% as compared with 19% in the GO1 group (HR, 0.727 [95% CI, 0.530-0.997]; *P* = .048). Survival benefit from GO2 in patients with mutated *IDH* was not significantly affected by the presence of secondary-type mutations (test for heterogeneity, *P* = .56; supplemental Figure 4B). There was also no detectable difference in the survival benefit from GO2 according to the presence or absence of secondary-type mutations in the overall cohort, excluding patients with adverse cytogenetics or *TP53* mutations (test for heterogeneity, *P* = .58; supplemental Figure 4C). For patients with *NPM1* mutations, the 5-year OS was 29% with GO2 vs 24% with GO1 (HR, 0.84; 95% CI, 0.58-1.20; *P* = .340).

### Treatment toxicity and resource usage

The frequencies and severities of early deaths and other adverse events were comparable between the 2 treatment arms (Table 2; supplemental Figure 5), including those relating to liver toxicity and deaths attributed to infection or hemorrhage. There was no significant difference between the 2 groups for the time of neutrophil and platelet recovery after course 1 (supplemental Table 2). The kinetics of blood count recovery between treatment arms were evaluated specifically for the clinical or genetic secondary AML subgroups. Of interest, platelet recovery times were longer after GO2 in patients with genetic secondary AML (median days, 32 [quartiles 27 and 40] vs 30 [quartiles 26 and 35] after GO1; *P* = .027).

With respect to supportive care, more platelet transfusion were required with GO2 (13 vs 11 days; *P* = .002), but units of blood, days of receiving antibiotics or hospitalization, or the time to the start of course 2 did not differ between GO2 and GO1 arms (supplemental Table 2).

### Outcome in patients who received transplantation

**Figure 5.**
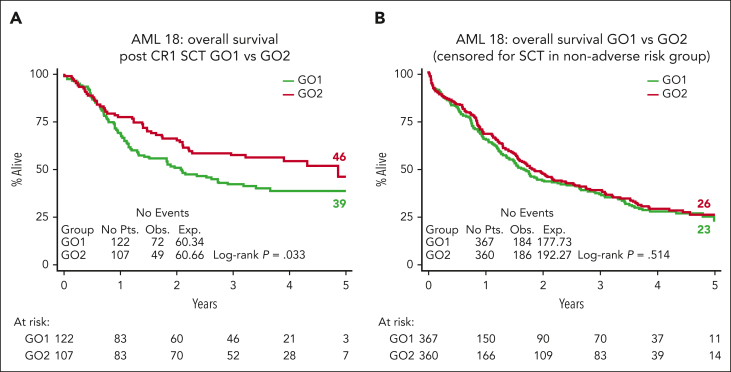
**Transplant-related survival outcomes.** (A) OS according to the treatment arm of patients who received an allogeneic stem cell transplantation in CR1, landmarked from the date of transplantation. The OS at 4 years from CR1 ASCT was 54% after GO2 induction vs 39% after GO1 (*P* = .021). (B) OS according to the treatment arm of patients without adverse cytogenetics or mutated *TP53* censored at ASCT.

**Figure 6.**
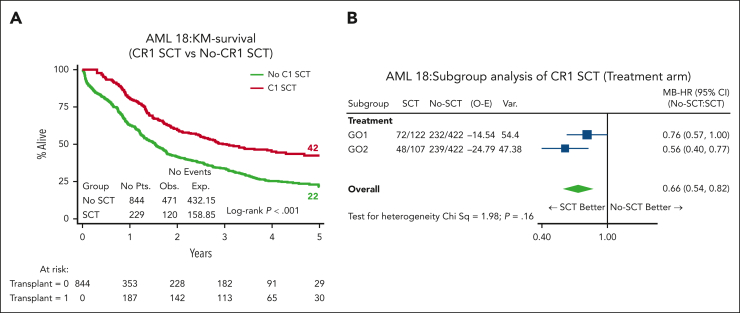
**Effect of transplant vs no transplant on survival.** (A) OS based on whether patients received ASCT by Mantel-Byar analysis. In the Mantel-Byar analysis, all patients started in the no transplant group and were transferred to the transplant group at the time of transplantation. (B) Mantel-Byar analysis for survival according to ASCT in CR1 by treatment arm. KM, Kaplan-Meier.

In total, 272 (32%) patients received an allogenic stem cell transplant (ASCT); most transplants (n = 229, 84%) were delivered in first remission (CR1) (27% of cohort; GO1, 122 and GO2, 107; Table 2). The median age of patients who received transplantation was lower (64.5 years vs 66 years; *P* = .061), and only 12 (GO1, 8 and GO2, 4) were performed among patients aged >70 years. Notably, OS and RFS from the time of transplant in CR1 was superior for patients in the GO2 arm compared with those in the GO1 arm (OS: HR, 0.67; 95% CI, 0.47-0.97; *P* = .033; Figure 5A; RFS: HR, 0.66; 95% CI, 0.46-0.93; *P* = .017; supplemental Figure 6). OS at 4 years from CR1 ASCT was 54% after GO2 induction vs 39% after GO1 (HR, 0.65; 95% CI, 0.45-0.91; *P* = .021). Additionally, the OS benefit from GO2 observed among the group without adverse cytogenetics or *TP53*-mutated AML (Figure 1) was lost when the data of patients were censored at ASCT (Figure 5B). Although there was a survival advantage for ASCT based on Mantel-Byar analysis in the overall cohort (HR, 0.66; 95% CI, 0.54-0.82; *P* < .001; Figure 6A), further investigation stratified according to the treatment arm showed that this benefit was most evident in patients who had received GO2 induction (Figure 6B).

## Discussion

This trial has explored the optimal scheduling of GO when administered with AML induction chemotherapy. The NCRI AML16 had previously shown an OS benefit from a single GO dose in older patients.^1^ However, the approved schedule, based upon the ALFA 0701 trial, uses a fractionated schedule, with 3 doses of GO given in course 1 and subsequent single doses given in courses 2 and 3.^2^ In this study, we used just 2 doses of GO in course 1 because of concerns of veno-occlusive disease risk and delayed blood count recovery; we also omitted GO beyond course 1 because the NCRI AML15 trial had shown no evidence of benefit in consolidation.^17^ Additional toxicity from this fractionated schedule compared with that from a single GO dose seems restricted to platelet recovery, most evident in patients with secondary AML–like mutations; there was no detrimental effect with regard to liver toxicity.

We excluded patients with known adverse risk cytogenetics from entry because there is no evidence that GO improves outcomes in this genetic subgroup.^4^ However, the randomized cohort included a significant number of patients with adverse karyotype because these patients were enrolled before their genetic results were available. Additionally, when applying the ALFA1200 genetic model groups, validated to predict outcomes from intensive chemotherapy specifically in older adults, this AML18 cohort had a similar proportion of patients categorized as being in the poorest risk no-go group to that of the reported ALFA 1200 cohorts.^10^^,^^18^ In view of this, it is not unexpected that in the total randomized cohort, there was no apparent significant survival benefit from GO2. However, in a sensitivity analysis that excluded patients with adverse cytogenetics and/or *TP53* mutations, a survival benefit for GO2 was seen, both overall and in patients who received transplantation in the first remission. This benefit of GO2 was associated with a clear correlation with leukemia clearance and CR without MRD (ELN response criteria^7^^,^^15^). The differential MRD-measured response for GO2 vs GO1 varied across molecular subtypes and was greatest for *IDH* mutations (MRD <0.1%; 71% GO2 vs 47% GO1 for *IDH2* and 76% GO2 vs 62% GO1 for *IDH1*), consistent with the observed survival advantage from GO2 in patients with *IDH* mutation. Interestingly, patients who achieved MRD negativity after GO2 had significantly improved survival compared with patients who had an MRD-negative status after GO1, implying a greater reduction in disease burden with GO2, even in patients nominally with undetectable MRD (by the flow cytometric sensitivity threshold of ∼10^–4^). That postinduction deeper remissions are predictive for outcomes after subsequent therapy is supported by the reduced relapse risk and survival benefit observed after CR1 transplantation in patients who received GO2 at induction. However, the loss of survival benefit of GO2 when survival was censored at transplantation underlines the importance of allograft in consolidating postinduction MRD-negative responses in older adults because their relapse risk remains high despite initial chemosensitivity.^14^^,^^19^ The benefit for reduced intensity transplantation in CR1 in older patients is consistent with our findings in the NCRI AML 16 trial,^20^ but, here, the 5-year survival approached 50% in patients receiving GO2 in induction and then a CR1 transplant. Although the transplant rate in this trial was higher than in our previous AML16 trial in this age group (29% vs 15%), not all patients are suitable, particularly patients aged >70 years, and this may explain the lack of detectable benefit from GO2 in patients aged >70 years. Disease biology in older patients may contribute to poorer outcomes through treatment resistance; however, we note that the improved leukemia clearance from GO2 was also evident in patients aged >70 years (supplemental Figure 7). Therefore, optimizing induction therapy for older patients remains important, particularly with the availability of oral azacytidine maintenance therapy studies, which demonstrate that MRD negativity before initiation of maintenance is a strong prognostic indicator of survival.^2^^,^^21^^,^^22^

In conclusion, this randomized trial demonstrates that a fractionated schedule of GO has superior efficacy for leukemia clearance to a single dose in older adults without adverse risk genetics and can be administered safely as 2 doses with the first course. This difference in induction efficacy can be converted to a significant longer-term survival benefit by allotransplantation in patients aged between 60 and 70 years.

Conflict-of-interest disclosure: S.D.F. declares research funding from Jazz and Bristol Myers Squibb; served on the speaker’s bureau of Jazz, Pfizer, and Novartis; and served on the advisory committee of MPAACT (Measurable residual disease Partnership and Alliance in Acute myeloid leukemia Clinical Treatment). P.V. declares honoraria from Celgene, Pfizer, Jazz Pharmaceuticals, AbbVie, Daiichi Sankyo, Astellas Pharma, and Celgene. P.M. declares honoraria from and serves on the speaker’s bureau of Pfizer, Jazz, AbbVie, and Astellas. S. Knapper. declares research funding from 10.13039/100004336Novartis; serves on the speaker’s bureau of Astellas, Novartis, and Jazz; declares consultancy with Servier and Bristol Myers Squibb. N.H.R. declares research funding from Jazz and 10.13039/100004319Pfizer and honoraria from Pfizer, Servier, and Astellas. The remaining authors declare no competing financial interests.

## Supplementary Material

Supplemental Tables, Figures, Methods, and References
