# Supplementary material for: Fractionated vs single-dose gemtuzumab ozogamicin with determinants of benefit in older patients with AML: the UK NCRI AML18 trial
Source: Blood. 2023 Aug 22;142(20):1697–707. doi: 10.1182/blood.2023020630 (PMC10667325; doi:10.1182/blood.2023020630)
Supplement: Supplemental Tables, Figures, Methods, and References [file BLOOD_BLD-2023-020630-mmc1.pdf]

Supplementary Table 1. Patient characteristics according to presence or absence of MRD data

	MRD data N=609	No MRD data N=235	P value
**Treatment Randomisation**			
GO1	307 (50%)	115 (49%)	0.701
GO2	302 (50%)	120 (51%)	
**Age** median (Qrange)	67 (51 – 81)	68 (50 – 79)	0.025
<65	179 (29%)	59 (25%)	0.046
65-69	241 (40%)	82 (35%)	
70+	189 (31%)	94 (40%)	
**Male**	373 (61%)	138 (59%)	0.501
**WBC** x 10^9 / L median (Qrange)	6 (0.4 – 416.6)	5.2 (0.3 – 198.2)	0.289
<10	353 (58%)	140 (60%)	0.830
10 – 49.9	184 (30%)	66 (28%)	
50+	72 (12%)	29 (12%)	
**Diagnosis**			
De Novo AML	494 (81%)	179 (76%)	0.257
Secondary AML	61 (10%)	28 (12%)	
High Risk MDS	54 (9%)	28 (12%)	
**Performance ID** (ECOG)			
0	312 (51%)	92 (39%)	<0.001
1	272 (45%)	114 (49%)	
2	25 (4%)	29 (12%)	
**Genetic risk**			
**Cytogenetic** (Grimwade2010)			
Favorable	24 (5%)	6 (3%)	0.727
Intermediate	415 (75%)	143 (73%)	
Adverse	73 (13%)	30 (16%)	
Unknown	40 (7%)	16 (8%)	
**TP53+**	45 (8%)	24 (12%)	0.085
**ALFA 1200**			
Go-go (Favorable)	203 (40%)	71 (40%)	0.234
Slow-go (Intermediate)	272 (53%)	88 (49%)	
No-Go (Unfavorable)	37 (7%)	20 (11%)	

Supplementary Table 2. Recovery Times and Resource Usage by Treatment Arm

	GO1	GO2	P value*
Neutrophil recovery Time from start of Course 1<br>Days, median [IQR]			
All patients	29 [26,35]	29 [26, 33]	0.348
Clinical Secondary AML	31 [27.5, 33.5]	30 [26, 35]	0.564
Genetic Secondary AML	32 [27, 37]	30 [28, 35]	0.972
Platelet recovery Time from start of Course 1<br>Days, median [IQR]			
All patients	29 [26,33]	30 [26,35]	0.074
Clinical Secondary AML	30 [26, 38]	31 [25, 35]	0.623
Genetic Secondary AML	30 [26, 35]	32 [27, 40]	0.027
Resource usage			
Units of blood	9 [6, 12]	9 [6, 13]	0.594
Units of platelets	11 [7, 16]	13 [8, 19]	0.002
Days of IV antibiotics	19 [12, 26]	20 [14, 26]	0.335
Days of oral antibiotics	7 [0, 18]	7 [0, 16]	0.782
Nights in hospital	33 [28, 40]	34 [28, 40]	0.937
Time to Course 2<br>Days, median [IQR]*	48 [43, 55]	48 [44, 56]	0.542

Legend. Log-rank test to compare the durations and Wilcoxon rank – sum (Mann–Whitney) test for comparing the number of units.
*for patients receiving course 2 on trial (GO1, 309; GO2, 304).
Abbreviations: IQR, interquartile range

Supplementary Figure Legends

Supplementary Figure 1.
Disposition of MRD data.

Supplementary Figure 2.
Subgroup analysis of overall survival for baseline characteristics according to treatment arm.

Supplementary Figure 3. Remission rates by molecular subtypes according to treatment arms, includes randomised patients without MRD data.

Supplementary Figure 4. A. Overall survival by Molecular Subtypes according to treatment arm
B. Overall survival in *IDH* mutated patients with or without secondary-type AML mutations according to treatment arm
C. Forest plot for OS in patients without adverse cytogenetics/mutated *TP53* by presence or not of secondary-type AML mutations

Key sAML, secondary AML

Supplementary Figure 5. Adverse Events (intention to treat population).
The percentage of patients with grades 1-5 events are shown for adverse events by treatment arm.
Inset table shows adverse event early deaths (day 60 deaths) by treatment arm

Supplementary Figure 6. Relapse free survival by treatment arm of patients who received an allogeneic stem cell transplantation in first remission, landmarked from the date of transplantation.

Supplementary Figure 7. Flow cytometric MRD levels in BM post course 1 according to treatment arm in patients ≥70yrs old. Median levels, presented as a percentage, were lower in GO2 arm (median, 25-75% quartiles with 1-99 percentiles are shown) although p value did not reach significance (P=0.0708 using the Mann-Whitney U test for continuous variables). Results represent all patients with MRD data (including those not in CR/CRi post course 1). Of patients ≥70yrs old without MRD data post course 1, 65.9% (27/41) and 56.6% (30/53) were refractory in GO1 and GO2 arms respectively.

Supplementary Figure 1. Disposition of MRD data

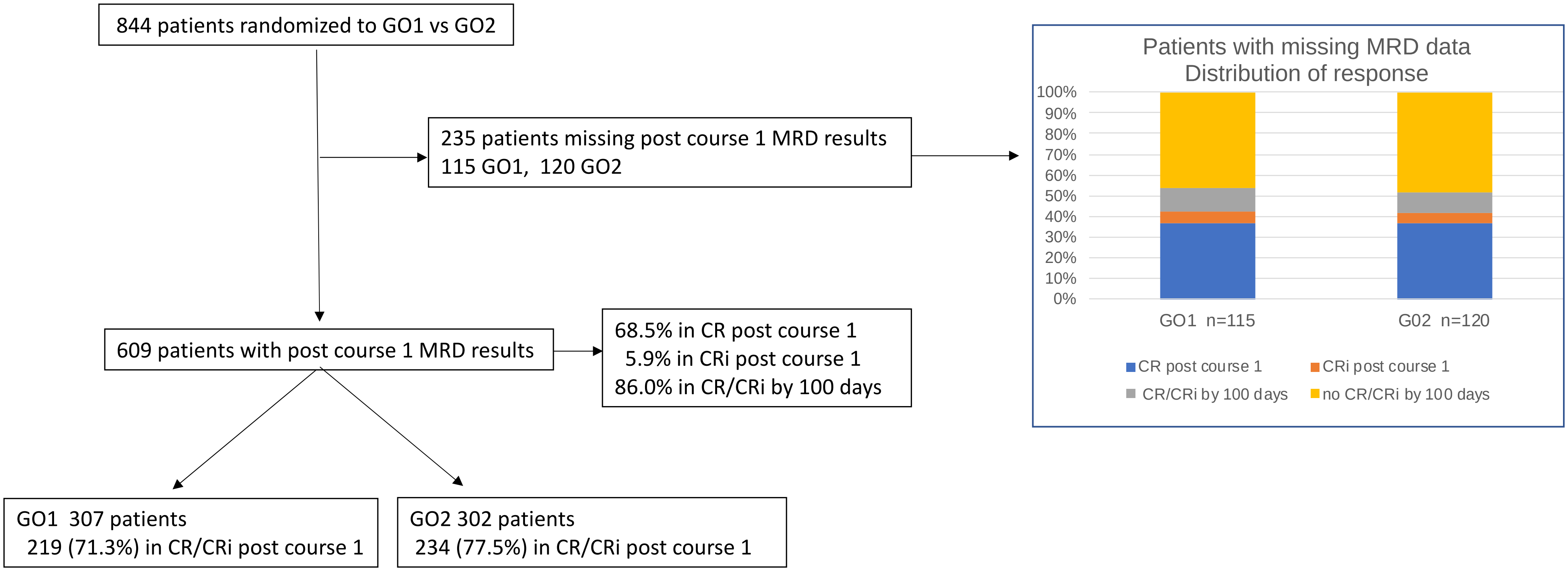

Supplementary Figure 2. Subgroup analysis of overall survival for baseline characteristics according to treatment arm.

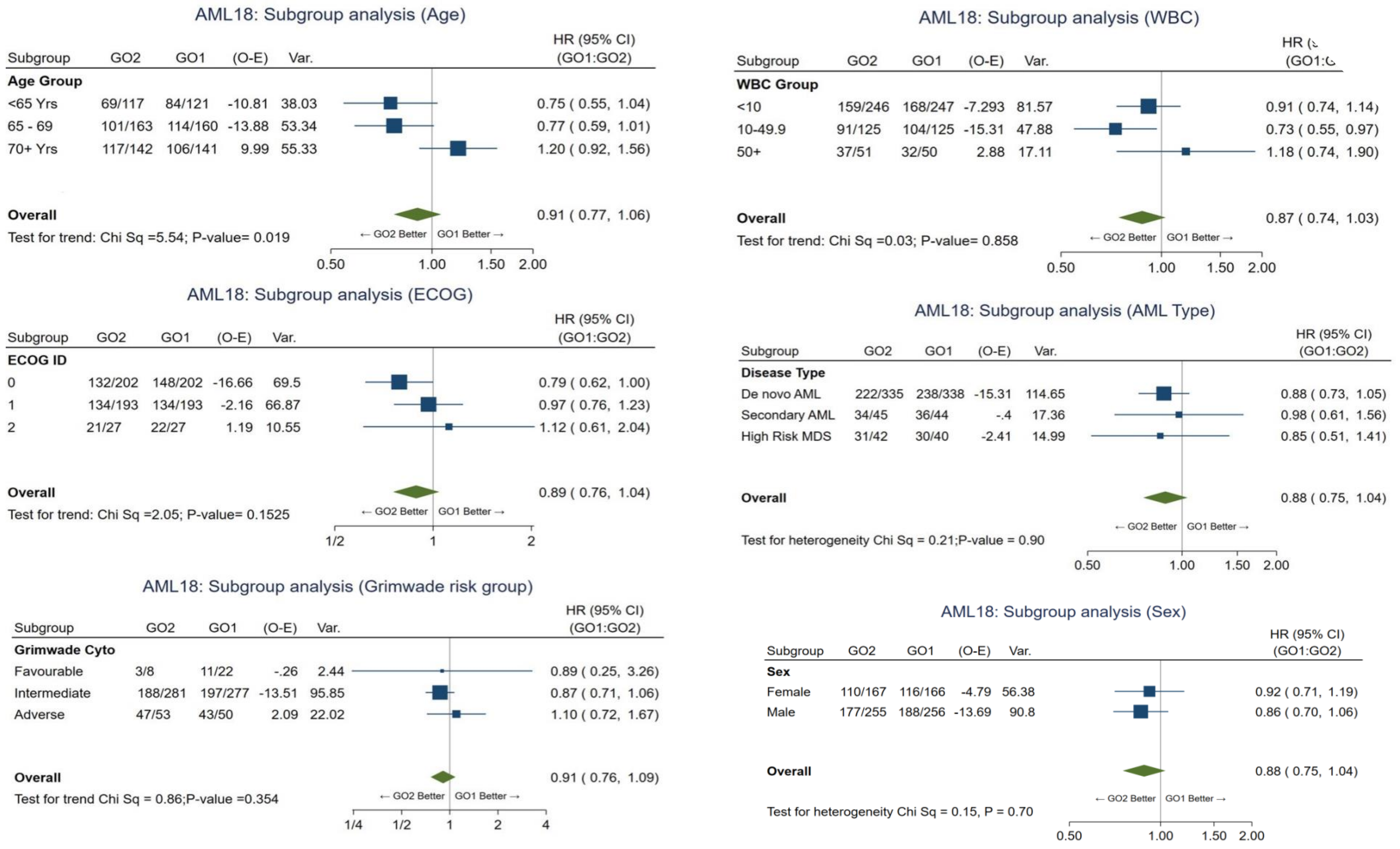

Supplementary Figure 3. Remission rates by Molecular subtypes

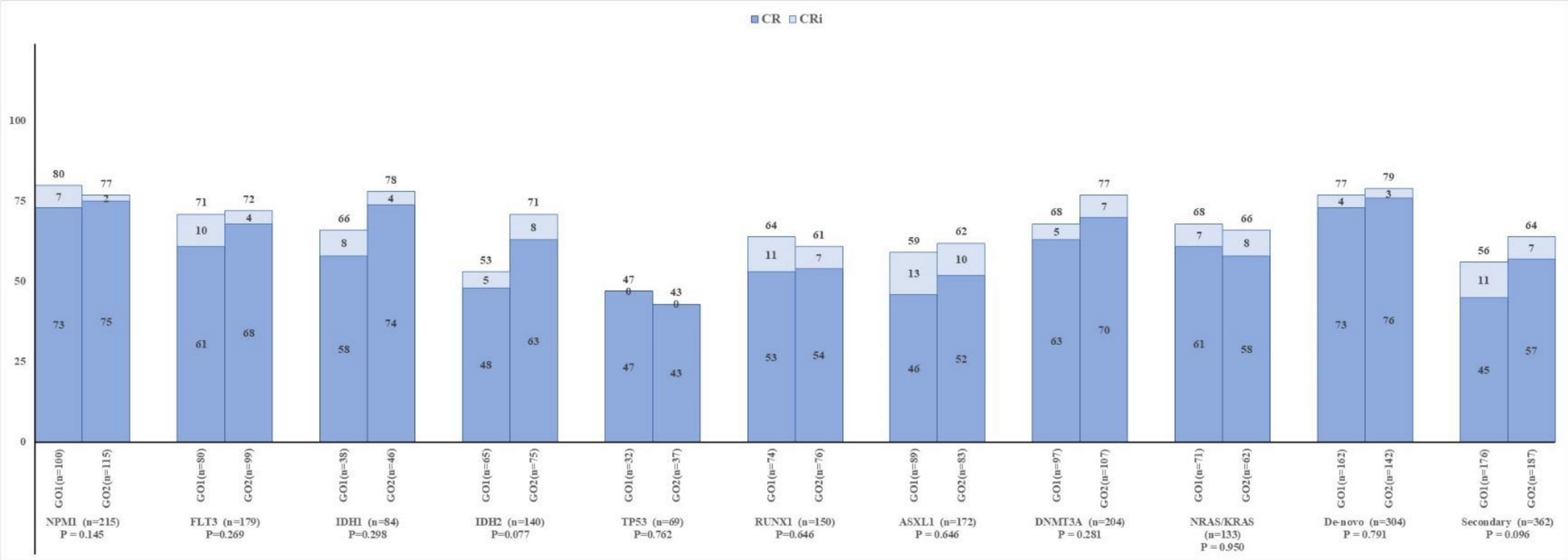

Supplementary Figure 4A

Forest plot for OS by molecular subtype

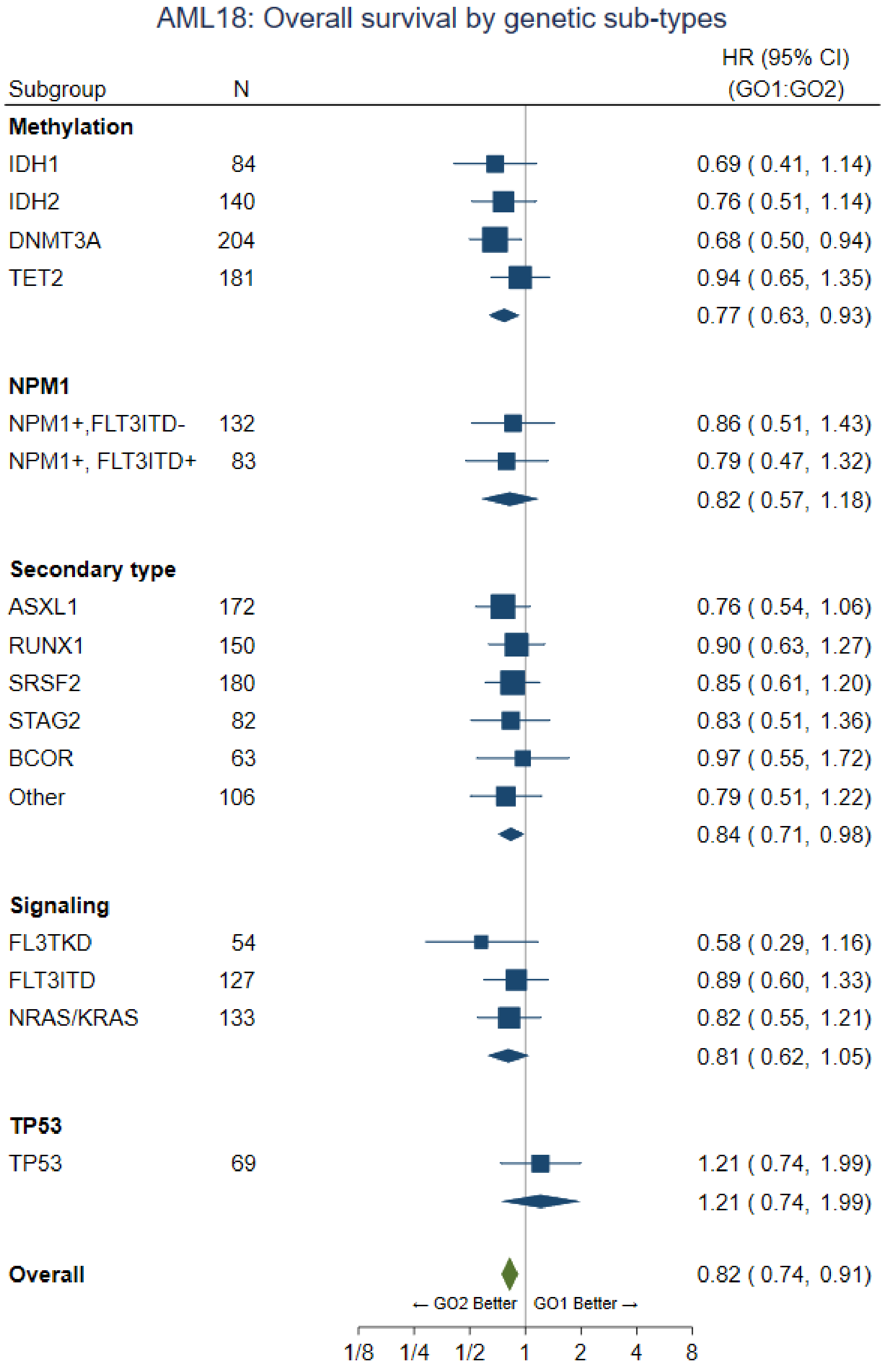

Supplementary Figure 4B Forest plot for OS in IDH mutated patients with or without secondary-type AML mutations

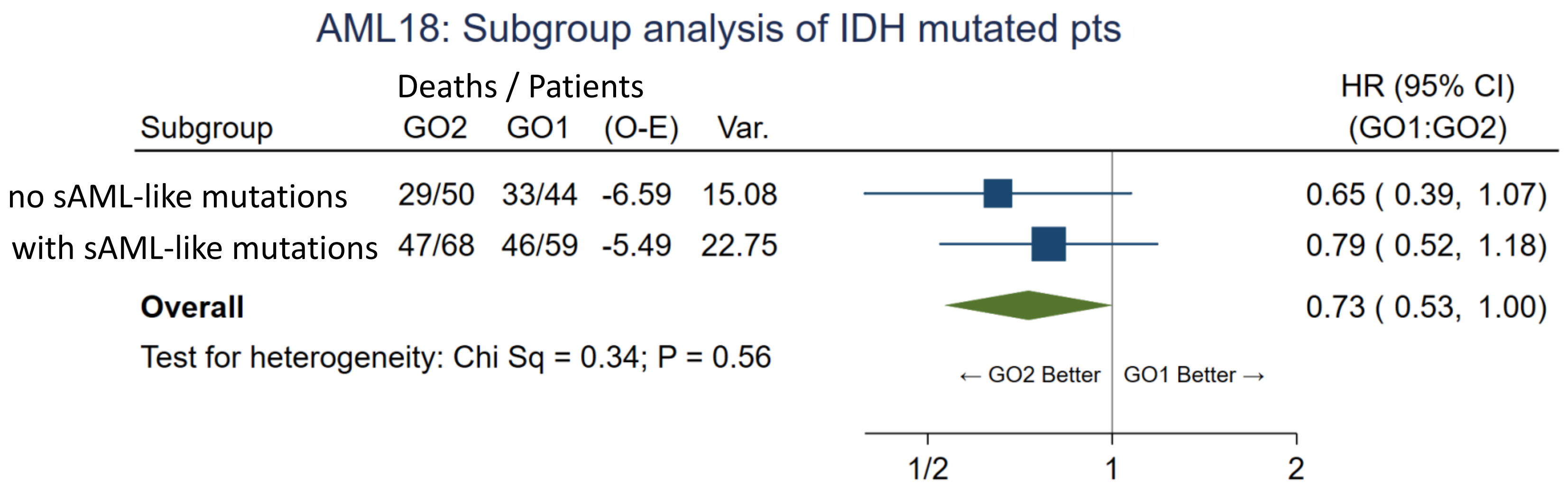

Supplementary Figure 4C Forest plot for OS in patients without adverse cytogenetics/TP53 by presence or not of secondary-type AML mutations

AML18: Subgroup analysis of mutation type (Non-adverse risk Pts)

Subgroup	Deaths / Patients GO2	Deaths / Patients GO1	(O-E)	Var.	HR (95% CI) (GO1:GO2)
**Mutation**					
with sAML-like mutations	123/170	128/163	-14.75	61.73	0.79 (0.62, 1.01)
no sAML-like mutations	79/141	95/156	-5.52	43.32	0.88 (0.65, 1.19)
**Overall**					0.83 (0.68, 1.00)

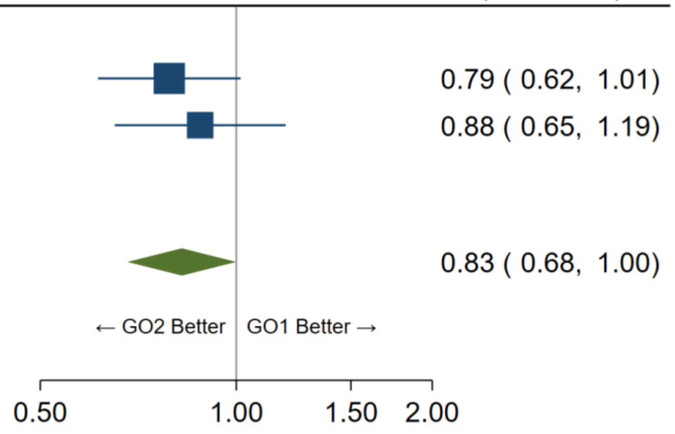

Test for heterogeneity Chi Sq = 0.30, P-value = 0.58

Supplementary Figure 5. Toxicities

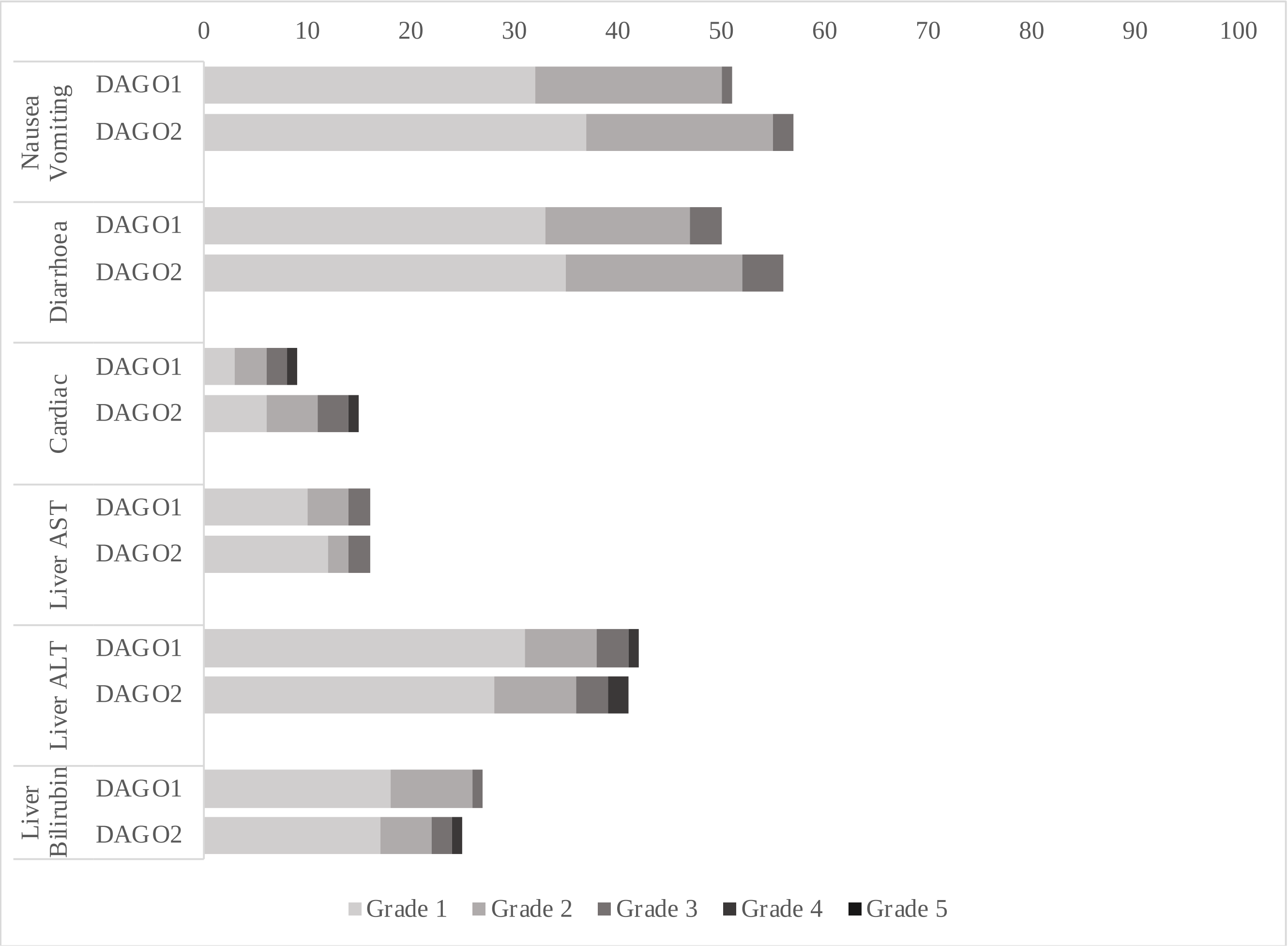

Deaths	GO1	GO2
Cause	(N)	(N)
Infection	22	20
Haemorrhage or CVA	5	3
VOD	0	1
Hepatic	0	1
Cardiac	3	2
Renal	1	0

Supplementary Figure 6.
Relapse free survival by treatment arm of patients who received an allogeneic stem cell transplant in first remission

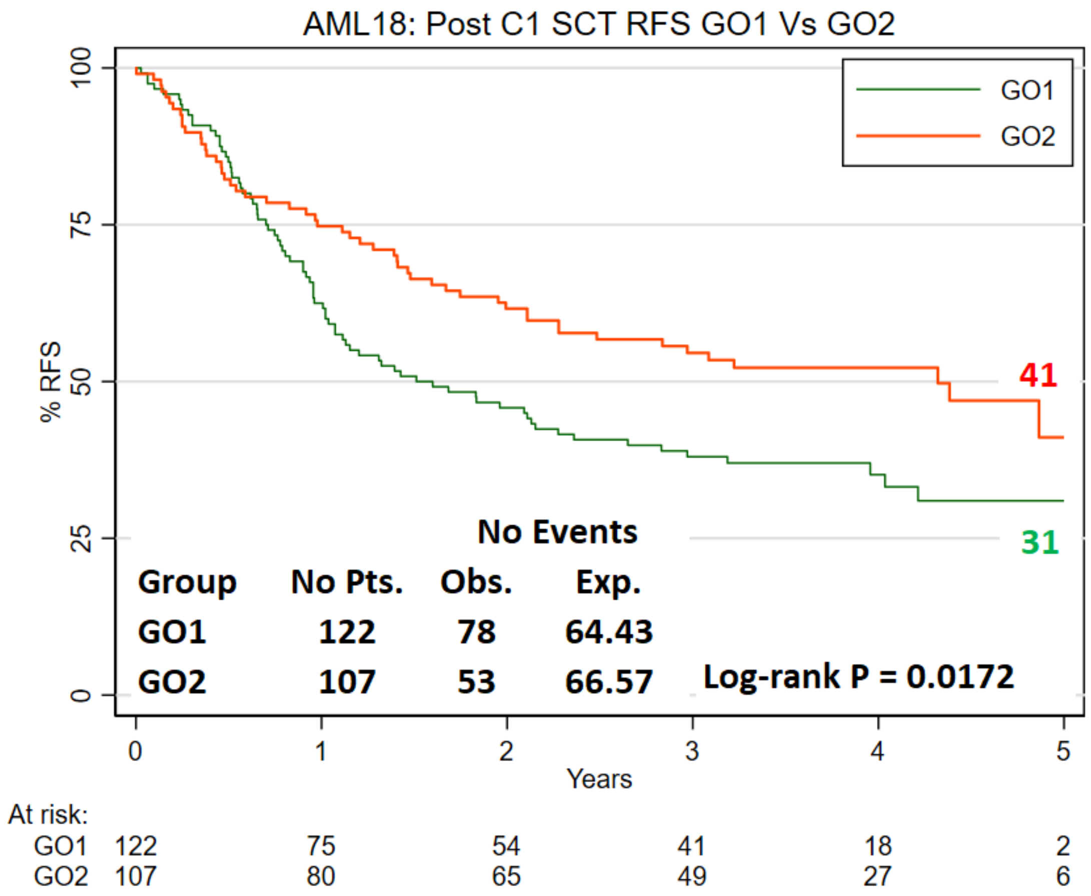

Supplementary Figure 7. Flow cytometric MRD levels in BM post course 1 according to treatment arm in patients ≥70yrs old.

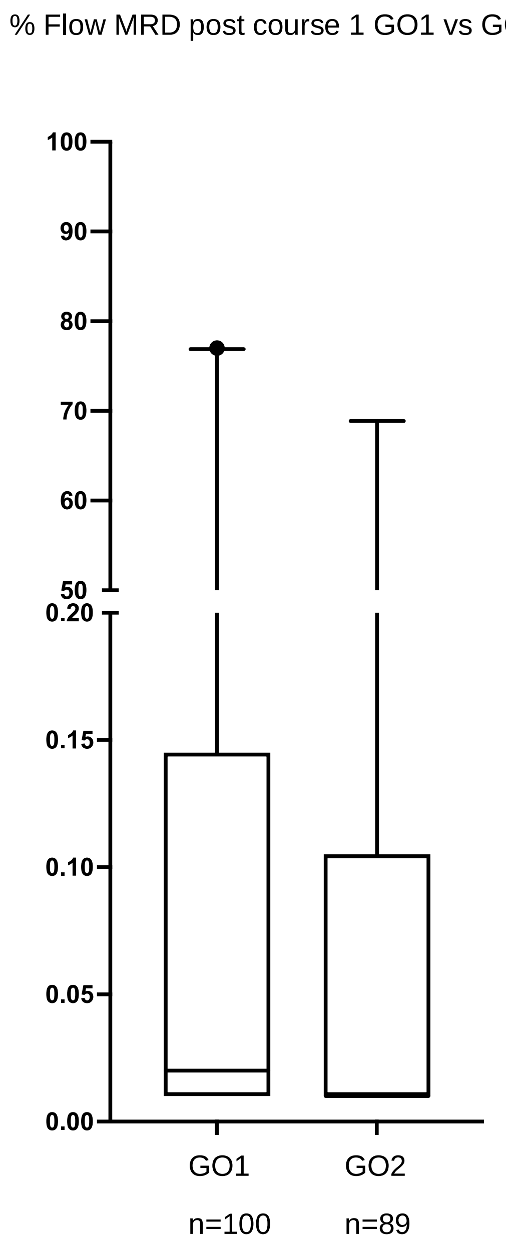

Supplementary Methods

Targeted sequencing

Mutational analysis was performed using a targeted panel of DNA capture probes designed to achieve full exonic coverage of 87 genes recurrently mutated in myeloid malignancies and hotspot coverage of an additional 10 genes[1-6]. DNA from peripheral blood or bone marrow mononuclear cells (MNCs) was extracted from baseline samples. Pre-capture DNA libraries were prepared using the KAPA HyperPlus protocol (KK8514, Roche) using a Biomek FXp Automated Workstation (Beckman Coulter). 100ng of genomic DNA was used as input for library preparation and amplified with 6 cycles of PCR according to the manufacturer's instructions. Targeted capture was performed using a custom pool of biotinylated capture probes (SeqCap EZ Prime Choice, Roche). Amplified DNA libraries were multiplexed in pools of 12 samples using 100ng of each library and hybridized to the capture probes according to the manufacturer's instructions. The captured DNA libraries were amplified by 14 cycles of PCR using a KAPA HiFi HotStart DNA polymerase and purified using AMPure XP Beads.

Post-capture amplified DNA libraries were quantified by Qubit (Life Technologies) and size distribution and quality analyzed using a Bioanalyzer chip (Agilent Technologies). Libraries were pooled in equimolar concentrations and were sequenced on an Illumina NextSeq 500 using 150 bp paired-end reads.

Variant calling in targeted panel sequencing

Sequencing data were analyzed in a custom pipeline based on the GATK Best Practices (GATK v4.1.2.0 and Picard v2.21.3). Raw sequencing reads were converted to an unmapped BAM file and adapter sequences soft-clipped using Picard MarkIlluminaAdapters. Following conversion back to a FASTQ file, reads were mapped to the hg38 human reference genome assembly using the Burrows–Wheeler aligner v0.7.17 [7] with alternate contig-aware alignment. Mapped and unmapped BAM files were merged using MergeBamAlignment and reads from different sequencing lanes were combined. Duplicate reads were marked using Picard MarkDuplicates and base quality scores recalibrated with GATK BaseRecalibrator and ApplyBQSR. Somatic variant calling was performed on the pre-processed BAM files using VarDictJava v1.7.0 [8] and Mutect2 [9] in tumour-only mode. For VarDict, variants were called with a minimum variant allele frequency of 0.01, minimum base quality score of 25 and minimum supporting reads of 2, with indel realignment and removal of adapter sequences. For Mutect2, a minimum tumor LOD of 1 was used, and variants were filtered for sequence context-dependent artefacts using FilterMutectCalls and FilterByOrientationBias. Indels were left-aligned and normalized using bcftools norm (v1.9). Variants were annotated using Annovar.[10] Target enrichment metrics and coverage was calculated using Picard CollectHsMetrics and only samples with a median target coverage >140x non-duplicate reads were used for downstream analysis.

VarDict and Mutect2 variant calls were analyzed separately to identify a consensus list of high-confidence variants. The following post-processing filters were applied to VarDict calls to exclude likely sequencing artefacts: (1) Minimum of 5 variant reads for SNVs (with at least 2 reads in forward and reverse directions), or minimum of 10 variant reads for indels (with at least 4 reads in forward and reverse directions). (2) Minimum base quality score 30. (3) Minimum mapping quality score 40, except for variants in *U2AF1;U2AF1L5*, where the mapping quality was ignored. This is because in hg38, there is a duplication of the *U2AF1* gene on chromosome 21 called *U2AF1L5*, which results in reads being flagged as multi-mapped. (4) No position bias towards beginning or end of reads. The following post-processing filters were applied to Mutect2 calls: (1) Passed all default Mutect2 filters or only failing one of the '*clustered_events', 'haplotype', 'germline' or 'slippage'* filters. 2) Minimum of

5 variant reads for SNVs (with at least 2 reads in forward and reverse directions), or minimum of 10 variant reads for indels (with at least 4 reads in forward and reverse directions).

Variants were flagged as likely germline or sequencing artefacts if any of the following applied: (1) Variants with a population allele frequency >1 in 1,000 according to any of three large polymorphism databases (Gnomad, 1000 Genomes Project, ESP6500) that is not a hotspot driver mutation with COSMIC (v88) recurrence >100 or is present in a list of clonal hematopoiesis-associated mutations compiled from five large studies. [3,4,6,11,12] (2) Variants detected ≥2 times in a panel of ~100 normal cord blood and bone marrow samples from healthy individuals. (3) Variants that were recurrent in ≥5% of the cohort unless previously reported as somatic in COSMIC (v88).

Finally, we kept non-synonymous exonic and splicing variants with a variant allele frequency ≥0.02 for the final analysis. Filtered variants from Mutect2 and VarDict were intersected, and variants called by both callers were retained. The remaining variants that were only called by one of the two callers were further inspected using the Integrated Genome Viewer (IGV) and only high confidence variants were included in the final variant list, matching any of the following criteria: (1) Known hotspots (previously reported in haematological malignancies in COSMIC >=5 times). (2) *FLT3* internal tandem duplications (ITDs) and *NPM1* 4 bp insertions. (2) Variants previously observed in other samples in our own datasets. (4) Variants with VAF of ≥5%.

To improve the detection of *FLT3* ITDs we additionally called variants using Pindel (v0.2.5b9) with minimum mutant reads set to 2. *FLT3* ITDs > 2% VAF that were not identified by Mutect2 or VarDict were additionally included. The vast majority (157/166; 94.6%) of patients with *FLT3*-ITD were already identified by Mutect2 or VarDict, whereas 9/166 (5.4%) were detected by Pindel only.

Multiparameter Flow Cytometry (MFC) detection of MRD

Patients' samples were sent by overnight mail to the reference laboratory. Following ammonium chloride lysis, nucleated cells of bone marrow (and /or blood at diagnosis) were labelled with antibody panel shown in Supplementary Methods Table 1 for flow cytometric MRD analysis as previously described[13-15]. The conventional AML MFC-MRD assay screened for abnormal immunophenotypic expression in 2 antibody combinations (tubes 1 & 2) containing ELN recommended markers. In addition, Tube 3 was applied to detect immunophenotypic LSC-type aberrancies (from CLL1/ CD45RA/ CD123 expression on CD34+CD38-CD19- cells) with assay detection threshold of 0.02%. Cell acquisition was performed on a FACSCanto (BD Biosciences) flow cytometer. Acquisition was set for 500,000 to 1 million cells or as many cell events as possible for MRD samples. Post-acquisition analysis of the flow cytometry data was performed (blinded to clinical data) using FlowJo software (Treestar Inc). Data review for analyses included periodically updated reference control bone marrow profiles. Viability, acquisition and autofluorescence artefact and hemodilution (by CD11b / CD13 myeloid maturation profile) were assessed in acquisition generated flow cytometry standard (FCS) data files. Results from tube 3 provided additional information to tubes 1-2 for MFC-MRD but were not quantitated as LSC separately in this study. CD33 expression profile was evaluated on global myeloid cells including monocytes in order to exclude interference to detection of aberrant immunophenotypes from potential loss of CD33 binding /expression resulting from CD33 splicing polymorphisms or possibly from GO. In the first 500 MRD samples, global low myeloid expression was infrequent and not increased in GO2 (5 of 248 samples) compared to GO1 (10 of 252 samples).

MFC-MRD analysis

Flow cytometric MRD testing combined detection of diagnostic leukemic aberrant immunophenotypes (LAIP) and different from normal aberrant immunophenotypes (DfN) as per consensus recommendations[16,17] with any measurable level of MRD considered positive (above sensitivity threshold of 0.02-0.05% of leukocytes). An MRD negative result required negativity in an adequate bone marrow by both DfN and LAIP analysis (prerequisite of LAIP target(s) identified at baseline).

Baseline LAIPs were selected from blast subpopulations in diagnostic samples (bone marrow and /or blood that deviated from the normal antigen profiles with sufficient detection sensitivity, usually comprised >10% of leukemic blasts and, from previous data[13,14,18] were known to be stable at follow-up (~0.02-0.05% sensitivity thresholds). Most LAIPs were defined by pre-set 'different-from normal' regions (gates) applied to CD117+ and CD34+ blasts (gated by FSC/SSC/CD45/ CD117 or CD34). This analysis approach was also applied to screen for any DfN aberrant immunophenotypes in all MRD samples including those with no baseline data. DfN pre-set gates were 'empty' for control bone marrow CD117+ or CD34+ blasts (empty defined as <10^{-4} mean+SD of >20 reference bone marrows). LAIP/DfN gates that included weak CD33 as a parameter were adjusted or excluded if myeloid CD33 expression was globally low. If LAIP/DfN gates included events that might result from background (including artefact from autofluorescence), backgating was performed to check distribution of events in other marker and light scatter profiles. When there were increased myeloid blasts but no LAIPs from 'different to normal' regions, CD117+ and/or CD34+ leukemic blasts were overlayed with reference controls ('normal' CD117+ and/or CD34+ blasts) to further check for aberrant immunophenotypes. If there was an expanded myeloid blast population that was mainly or all negative for CD117 and CD34, blasts were gated by CD45/SSC or FSC/SSC then CD45intermediate and other markers (such as HLADR, CD56, CD33, CD13) followed by overlaying with reference controls to identify LAIPs for which sensitivity threshold was at least 0.05% of leukocytes. Potential LAIPs that overlapped with mature monocyte profiles (usually because of higher CD45 expression) were not reported as MRD unless these predominated in patients with clear refractory disease by flow cytometry. Our panel was insufficiently comprehensive to discriminate monocytic LAIPs for MRD sensitivity.

MRD percentages were reported as percentage of leukocytes (CD45+) expressing the identified blast LAIP with the highest frequency and/or specificity and stability[16,17]. Any level of MRD detected above the sensitivity threshold for a baseline defined LAIP was reported as MRD positive in AML18. MRD positivity was reported by different from normal approach if above sensitivity threshold (0.02%-0.05%, or >0.1% when increased background below this level from autofluorescence / viability artefact) in the pre-set different-from-normal gates. In some patients minor or major immunophenotypic changes from baseline or previous MRD sample LAIPs were detected but reported as MRD if fulfilled criteria for 'different-from-normal' approach. Samples were not reported if poor viability and/or fluorescent artefact with unacceptable background. Inadequate follow-up samples defined by <0.1% blasts and/or <100 cell events within the total blast (gated by CD45/SSC plus CD34+ and/or CD117+ gate) were also excluded from data analysis unless there was detectable MRD from a distinct aberrant cluster of at least 30-50 LAIP cell events.

Supplementary Methods Table 1. Flow cytometric MRD Antibody Panel

Tube No.	FITC	PE	PerCP	PECy7	APC	APC H7	BV 510	BV 421
1	**HLADR**<br>L243 (BD)	**CD13**<br>L138 (BD)	**CD34**<br>8G12 (BD)	**CD117**<br>1042D2 (BD)	**CD33**<br>P67.6 (BD)	**CD45**<br>2D1 (BD)	**CD14**<br>SJ25C1 (BD)	**CD11b**<br>ICRF44 (BD)
2	**CD38**<br>HB7 (BD)	**CD56**<br>MY31 (BD)	**CD34**	**CD117**	**CD33**	**CD45**	**CD19**<br>(Biolegend)	**CD7**<br>M-T701 (BD)
3	**CLL1**<br>(CLEC12A, BD)	**CD123**<br>7G3 (BD)	**CD34**	**CD117**	**CD19**<br>SJ25C1 (BD)	**CD45RA**<br>HI100 (BD)	**CD45**<br>HI30 (BD)	**CD38**<br>HIT2 (BD)

BD – Becton Dickinson Biosciences, Oxford, United Kingdom
BD Pharmingen – Becton Dickinson Biosciences - Pharmingen, Oxford, United Kingdom
Dako - Dako Agilent Technology

References

1. Cancer Genome Atlas Research N. Genomic and epigenomic landscapes of adult de novo acute myeloid leukemia. *N Engl J Med*. 2013;368(22):2059-2074.
2. Papaemmanuil E, Gerstung M, Bullinger L, et al. Genomic Classification and Prognosis in Acute Myeloid Leukemia. *N Engl J Med*. 2016;374(23):2209-2221.
3. Jaiswal S, Fontanillas P, Flannick J, et al. Age-related clonal hematopoiesis associated with adverse outcomes. *N Engl J Med*. 2014;371(26):2488-2498.
4. Genovese G, Kahler AK, Handsaker RE, et al. Clonal hematopoiesis and blood-cancer risk inferred from blood DNA sequence. *N Engl J Med*. 2014;371(26):2477-2487.
5. Xie M, Lu C, Wang J, et al. Age-related mutations associated with clonal hematopoietic expansion and malignancies. *Nat Med*. 2014;20(12):1472-1478.
6. Acuna-Hidalgo R, Sengul H, Steehouwer M, et al. Ultra-sensitive Sequencing Identifies High Prevalence of Clonal Hematopoiesis-Associated Mutations throughout Adult Life. *Am J Hum Genet*. 2017;101(1):50-64.
7. Li H, Handsaker B, Wysoker A, et al. The Sequence Alignment/Map format and SAMtools. *Bioinformatics*. 2009;25(16):2078-2079.
8. Lai Z, Markovets A, Ahdesmaki M, et al. VarDict: a novel and versatile variant caller for next-generation sequencing in cancer research. *Nucleic Acids Res*. 2016;44(11):e108.
9. Cibulskis K, Lawrence MS, Carter SL, et al. Sensitive detection of somatic point mutations in impure and heterogeneous cancer samples. *Nat Biotechnol*. 2013;31(3):213-219.
10. Yang H, Wang K. Genomic variant annotation and prioritization with ANNOVAR and wANNOVAR. *Nat Protoc*. 2015;10(10):1556-1566.
11. Abelson S, Collord G, Ng SWK, et al. Prediction of acute myeloid leukaemia risk in healthy individuals. *Nature*. 2018;559(7714):400-404.
12. Desai P, Mencia-Trinchant N, Savenkov O, et al. Somatic mutations precede acute myeloid leukemia years before diagnosis. *Nat Med*. 2018;24(7):1015-1023.
13. Freeman SD, Hills RK, Virgo P, et al. Measurable Residual Disease at Induction Redefines Partial Response in Acute Myeloid Leukemia and Stratifies Outcomes in Patients at Standard Risk Without NPM1 Mutations. *J Clin Oncol*. 2018;36(15):1486-1497.
14. Freeman SD, Virgo P, Couzens S, et al. Prognostic relevance of treatment response measured by flow cytometric residual disease detection in older patients with acute myeloid leukemia. *J Clin Oncol*. 2013;31(32):4123-4131.
15. Craddock C, Jackson A, Loke J, et al. Augmented Reduced-Intensity Regimen Does Not Improve Postallogeneic Transplant Outcomes in Acute Myeloid Leukemia. *J Clin Oncol*. 2021;39(7):768-778.
16. Heuser M, Freeman SD, Ossenkoppele GJ, et al. 2021 Update on Measurable Residual Disease (MRD) in Acute Myeloid Leukemia (AML): A Consensus Document from the European LeukemiaNet MRD Working Party. *Blood*. 2021.
17. Schuurhuis GJ, Heuser M, Freeman S, et al. Minimal/measurable residual disease in AML: a consensus document from the European LeukemiaNet MRD Working Party. *Blood*. 2018;131(12):1275-1291.
18. Bradbury C, Houlton AE, Akiki S, et al. Prognostic value of monitoring a candidate immunophenotypic leukaemic stem/progenitor cell population in patients allografted for acute myeloid leukaemia. *Leukemia*. 2015;29(4):988-991.

The following investigators recruited patients:

Aalborg Hospital, Marianne T Severinsen, Anne Roug, Gitte Madsen, **Aarhus University Hospital**, Hans Beier Ommen, **Aberdeen Royal Infirmary**, Dr D J Culligan, Dr J Tighe, Dr Gavin Preston, Dr M Lamaechia, Manmeet Randhawa, Dr A. Lawrie, Thura Win Htut, Wail Abdelrahman, **Addenbrooke's Hospital**, Dr Faisal Basheer, Charles Crawley, Ben Uttenthal, Michael Chapman, Dr Kiran Tawana, **Aintree University Hospital**, Dr Vikram Singh, Dr Lynny Yung, Dr Jeffery Smith, Dr Vikram Singh, Dr Indrani Karpha, Dr Katherine Lindsay, **Arrowe Park Hospital (Wirral)**, Dr Ranjit Dasgupta, Dr Elizabeth Jones, Dr Barbara Hammer, Dr Giridharan Durgam, **Basingstoke and North Hampshire Hospital**, Dr Henna Wong, Harberth Fernandez-Leyva, Henna Wong, Nigel Sargant, Benjamin Gray, Ashok Roy, Kanchana De Abrew, Holly Gilbert, Hussain Janan, **Beatson General Hospital (Gartnavel General Hospital)**, Professor Mhairi Copland, Pam McKay, Edward Fitzsimons, Mark Drummond, David Irvine, Anne Parker, Richard Soutar, Mike Leach, Nicholas Heaney, Grant McQuaker, **Belfast City Hospital**, Dr C Arnold, Dr R Cuthbert, Dr C Arnold, Dr D Finnegan, Dr Bethany Mitchell, Stephen Boyd Dr N.Cunningham, Rachel Brockbank, **Birmingham Heartlands Hospital**, Dr Vidyha Murthy, Shankara Paneesha, Richard Lovell, Bhuvan Kishore, Charalampos Kartsios, **Blackpool Victoria Hospital**, Dr Paul Cahalin, Dr Seye Kolade, Dr. Raisa Guerrero, Dr Bosko Andjelic, Johnathan Elliot, Naeem Desai, Ahmed Abdulgawad, Thomas Seddon, **Bradford Royal Infirmary**, Dr Suresh Krishnan, Dr Anshu Garg, Dr Adrian Williams, Nandini Sadasivam, Abida Naeem, **Bristol Haematology & Oncology Centre**, Dr Priyanka Mehta, Dr Rachel Protheroe, Dr Sanne Lugthart, **Castle Hill Hospital**, Dr Simone Green, Dr Andrew Fletcher, Dr Senthilkumar Durairaj, **Cheltenham General Hospital**, Dr A Rye, Dr Richard Lush, Dr Asha Johny, Dr Rebecca Frewin, Dr Michael Shields, Dr Adam Bond, Dr R McCulloch, **Christie Hospital**, Dr Mike Dennis, Dr Mohd Mamat, Dr Dan Wiseman, **Churchill Hospital**, Prof Paresh Vyas, Andy Peniket, **Clatterbridge Cancer Centre (Royal Liverpool)**, Dr Arpad Toth, Dr Floisand, Countess Of Chester Hospital Dr Arvind Pillai, Dr Salaheddin Tueger, Dr Gillian Brearton, **Crosshouse Hospital (Ayrshire and Arran Health Board)**, Dr W Gordon, Peter Maclean, Mark MacColl, Paul Micallef Eynaud, Ian Devanny, Lea Haskins, Mark Rafferty, **Derby Teaching Hospital**, Dr I Amott, Dr A Glover, Adrian Smith, Juanah Addada, Tom Taylor, Firas Al-Kaisi, Malik Saeed, **Derriford Hospital (Plymouth)**, Dr Patrick Medd, Simon Rule, Claire Hutchinson, Wayne Thomas, Tim Nokes, David Lewis, Sophie Johns, Rory McCulloch, Loredana Mihailescu, Sarah Mant, Gerorgina Symes, **Forth Valley Hospital**, Dr Hugh Edwards, Dr Rachel Boulton-Jones, Dr Katrina Farrell, Dr Sarah Barcroft, Dr. Hanlon, Dr R F Neilson, **Freeman Hospital (Newcastle)**, Dr Gail Jones, Dr Nicola Redding, Prof Graham Jackson, Dr Tobias Menne, Dr Wendy Osborne, Dr Erin Hurst, Dr Andrew Charlton, Dr Helen Marr, Dr Michelle Lannon, Dr Andrew McGregor, Dr Tom Fail, **Gloucestershire Royal Hospital**, Dr Adam Rye, Dr Richard Lush, Dr Asha Johny, Dr Rebecca Frewin, Dr Mike Shields, Dr Philip Robson, Dr Sally Chown, **Guy's Hospital**, Dr Richard Dillon, **Herlev University Hospital**, Claudia Schöllkopf, Mette Borg Clausen, Daniel El Fassi, Syed Azhar Ahmad, Bo Kok Mortensen, Nina Toft, **Hillingdon Hospital**, Dr Richard Kaczmarski, Dr Taku Sugai, Dr Akila Danga, Dr Ketan Patel, Dr Katherine Bailey, **Ipswich Hospital**, Dr Mahesh Prahladan, Dr Debo Ademokun, Dr Andrew Hodson, Dr Isobel Chalmers, Dr Ioana Whalley, **James Cook University Hospital**, Dr R Dang, Dr Diane Plews, Dr Jamie Maddox, Dr Angela Wood, Dr Marianna David, Dr Mohsen Norouzi, **Kettering General Hospital**, Dr Mark Kwan, Dr Jag Gandla, Dr Isaac Wilson-Morkeh, Dr AURANGZEB RAZZAK, Dr Rebecca Allchin, Dr Alex Gebreyes, Dr Matthew Player, **Leeds Teaching Hospital NHS Trust (St James)**, Dr Richard Kelly, Dr Anjum Khan, Dr Manish Jain, Prof David Bowen, Melvish Ul-Haq, **Leicester Royal Infirmary**, Dr Katherine Hodgson, Dr Murray Martin, Anna Tsoulkani, **Lincoln County Hospital**, Dr Charlotte Kallmeyer, Dr Osama Ali, Dr Gamal Sidra, Dr Ali Boden, Lauren Henry Dr Pavel Chudakou Dr Rajendra Pol, **Maidstone Royal Infirmary**, Dr Evangelia Dimitriadou, Dr Lalita Banerjee, Dr Richard Gale, Dr Clare Wykes, Adrian Maraj, **Manchester Royal Infirmary**, Dr Eleni Tholouli, Dr Fiona Dignan, Dr Sarah Burns, Caroline Wall, Margarita

Triantafillou, Thomas Seddon, Srividhya Senthil, Ahmed Amer, **Milton Keynes University Hospital**, Dr Moez Dungarwalla, Dr Subir Mitra, Dr Sarah Davis, Dr Magbor Akanni, **Musgrove Park Hospital (Taunton & Somerset Hospital)**, Dr Deepak Mannari, Simon Bolam, Belinda Austen, Lisa Lowry, **New Cross Hospital**, Dr Richard Whitmill, Dr Suprahk Basu, Dr Richard Whitmill, Dr Sophie Lee, **New Victoria Hospital**, Dr Gail Loudon, Dr Ian MacDonald, Dr Alistair Hart, **NHS Lanarkshire (Monklands, Wishaw and Hairmyres Hospital)**, Dr Christopher McDermott, Dr Linsay Mitchell, Dr Pamela Paterson, Dr Iain Singer, Dr Adrew Fyfe, Dr Charlotte Thomas, Dr Jane Laird, Dr Alice Cooke, Dr Annielle Hung, **Ninewells Hospital (Dundee)**, Dr Sudhir Tauro, Gordon Marron, Duncan Gowans, Raed-Alkhatib, Michelle Harrison, **Norfolk and Norwich University Hospital**, Dr Angela Collins, Dr Matthew Lawes, Prof Kris Bowles, Dr Nimish Shah, Dr Charlotte Hellmich, Dr Cesar Gomez, Dr Joel Cunningham, Victoria Willimott, **Northampton General Hospital**, Dr Jane Parker, Dr Sajjan Mittal, Dr Alistair McGrann, **Nottingham University Hospital**, Dr Jenny Byrne, Gerardo Errico, Jyoti Nangalia, Ruth Witherall, Prof Nigel H Russell, **Pinderfields General Hospital**, Dr Joanna Haughton, Dr David Wright, Dr John Ashcroft, Dr Kavita Patil, Dr Joanna Haughton, Dr Clare Kane, Dr William Wong, Dr Paul Moreton, Dr Muhammad Mohsin, **Poole General Hospital**, Dr Darshayani Furby, Dr Ram Jayaprakash, Dr Rebecca Maddams, Dr Louise Fraser, Dr Alistair Smith, Dr Bhoomika Roomalah, **Queen Alexandra Hospital (Portsmouth)**, Dr R Corser, Mary Ganczakowski, Robert Corser, Robert Ayto, Charle Alderman, Kanchana DeAbrew, Dr Gwynn Matthias, Emily Robinson, Dr Edward Belsham, **Queen Elizabeth Hospital Birmingham**, Vidhya Murphy, Dr Manos Raghavan, Malahat Saeed, Richard Buka, Dr Himabindu Rebbapragada, Dr Justin Loke, Dr Clare Lodwick, Dr Phillip Nicolson, **Queen's Hospital, Romford**, Dr Paul Greaves, **Raigmore Hospital**, Dr Caroline Duncan, Dr Jo Craig, Dr Peter Forsyth, Dr Katherine Leighton, Dr Frances Buckley, **Rigshospitalet University Hospital**, Ulrik Malthe Overgaard, Ove Juul Nielsen, Kim Theilgaard-moench, Kirsten Grønbæk, Peter Kampmann, Eva Funding, Eva Leinøe, Dr Lars Kjeldsen, **Roskilde Hospital**, Peter Møller, Morten Krogh Jensen, **Royal Bournemouth General Hospital**, Dr David Allotey, Renata Walewska, Helen McCarthy, Kunaal Kaushik, Gavinda Sangha, Rachel Hall, David Allotey, Dr Ifraz Hamid, Marcin Lubowiecki, **Royal Cornwall Hospital (Treliske)**, Dr Bryson Pottinger, Dr David Tucker, Ruth Witherall, Elizabeth Parkins, Adam Forbes, **Royal Devon & Exeter Hospital**, Dr Thomas Coates, Dr Jackie Ruell, Dr Paul Kerr, Dr Loretta Ngu, Dr Anthony Todd, **Royal Free Hospital**, Dr P Kottaridis, Dr Kate Cwynarsky, Dr Chris McNamara, Dr Mallika Sekhar, Dr Ashu Wechalekar, **Royal Marsden Hospital**, Dr David Taussig, Dr Mark Ethell, Dr Mike Potter, Dr Chloe Antheas, Nma-Okike Nzekwu, Dr Emma Nicholson, Dr Thinzar Ko Ko, Dr Patrick Elder, **Royal Oldham Hospital**, Dr David Osborne, Dr Allameddine Allameddine, Dr Hayley Greenfield, Dr Martin Rowlands, Dr Antonina Zhelyazkova, Dr Choudhuri Satarupa, Dr Atanas Stanchev, Dr Muhammad Pervaiz, **Royal Stoke University Hospital**, Dr Srivinas Pillai, Dr Richard Chasty, Dr Neil Phillips, Dr Kamaraj Karunanithi, **Royal United Hospital Bath**, Dr Chris Knechtli, Dr Sarah Wexler, Dr Josephine Crowe, Dr Sally Moore, Dr Gihan Mahmoud, Dr Rhys Williams, Dr Joanna Collins, **Russells Hall Hospital**, Dr Rupert Hipkins, Dr Stephen Jenkins, Dr Craig Taylor, Dr Jeff Neilson, Dr Avio Fernandes, Dr Ovine Gamage, Dr Yadanar Lwin, **Salford Royal Hospital**, Dr Rowena Thomas-Dewing, Dr Simon Jowitt, Dr Clare Barnes, Dr Mark Henry, **Salisbury District Hospital**, Dr Jonathan O Cullis, Dr Effie Grand, Dr Sally Bugg, Dr Tracey Parker, Dr James Milnthrope, **Sandwell Hospital**, Dr Farooq Wandroo, Dr Yasmin Hasan, Dr Richard Murrin, Dr Shivan Pancham, Dr John Gillson, Dr Wright, Dr Pancham, Dr Hisam Siddiqi, Dr S Altaf, **Singleton Hospital (Swansea)**, Dr Unmesh Mohite, Dr Hamdi Sati, Dr Hamid Majid, Dr Rhian Jones, **Southampton General Hospital**, Dr D Richardson, Dr Kim Orchard, Dr Matthew Jenner, Dr Srinivasan Narayanan, Dr Christopher Dalley, Dr Thomas Cummin, Dr Josh Dmochowski, **St Bartholomew's Hospital**, Dr Heather Oakervee, Dr Jamie Cavenagh, **St Helier Hospital**, Dr Simon Stern, Dr Caroline Ebdon, Dr Roslin Zuha, Dr Sneha Muthalali, Dr Stella Appiah-Cubi, Dr Corinne De Lord, Dr Emily Bart-Smith, **St Richard's Hospital**, Dr Santosh Narat, Dr Jamie Wilson, Dr Heba Yassin, Dr Salah Alhassan, **Sunderland Royal Hospital**, Dr Scott Marshall, Dr Victoria Hervey, Dr

Yogesh Upadhye, Dr Annete Nicolle, Dr Susanna Mathew, Dr Emily Graves, Dr Alexander Langridge, Dr Shikha Chattree, **Torbay District General Hospital**, Dr Rui Zhao, Dr Loredana Mihailescu, Dr Heather Eve, Dr Patrick Roberts, Dr Rui Zhao, Dr Barry Jackson, **University College London Hospital**, Dr Rob Sellar, Dr Vicky Stables, Wei Yee Chan, William Townsend, Suzanne Arulogun, Catriona Mactier, Nisha de Silva, **University Hospital Odense**, Duruta Weber, Claus Werenberg Marcher, Andreja Dimitrijevic, Klas Raaschou-Jensen, Maria Kallenbach, Mette Levring, **University Hospitals Coventry and Warwickshire**, Dr Beth Harrison, Dr Benjamin Bailiff, Dr Louise Fryearson, Dr Jhansi Muddana, Dr E Tebbet, Dr R. Afghan, Dr K. Randall, Dr Dunca Murray, Dr Anton Borg, Dr Francesca Jones, Dr Sarah Nicolle, Dr Salama Abosaad, Dr Maria Mushkbar, **University Hospital of Wales (Cardiff)**, Dr S Knapper, Dr Jonathan Kell, Dr Caroline Alvares, **Western General Hospital (Edinburgh)**, Dr Vicotria Campbell, Dr Huw Roddie, **Wexham Park Hospital**, Dr Mark Offer, Dr Simon Moule, Dr Nicola Bienz, Dr Nicola Philpott, Dr Carolina Lahoz, **Whiston & St Helens Hospital**, Dr Toby Nicholson, Dr J Tappin, Dr David Taylor, Dr Sammy Fergiani, Eleana Loizou, Ushma Meswani, Sally Evans, **Worcestershire Acute Hospitals NHS Trust**, Dr Nicholas Pemberton, Dr Clare Rowan, Dr Juliet Mills, Dr Safia Jalal, Dr Thomas Skibbe, Dr Iman Quereshi, Dr Khin Cho Thein, Dr Tracey Chan, **Worthing Hospital**, Dr. Santosh Narat, Andrew Wood, Dr Ronwyn Cartwright, Dr Santosh Narat, **York Hospital**, Dr Muhammad Naveed, Dr Lee Bond, Dr Jennifer Shields, Dr Laura Munro, Dr Annika Whittle, **Ysbyty Glan Clwyd**, Dr Earnest Heartin, Dr Yvonne Jones, Dr Margaret Goodrick, Dr Steph Jackson, Dr Durgadevi Moratuwagama, **Ysbyty Gwynedd**, Dr Ernest Heartin, Dr Chris Williams, Dr David Edwards
